# Structural, CSD, Molecular Docking, Molecular Dynamics, and Hirshfeld Surface Analysis of a New Mesogen, Methyl-4-(5-(4-(octyloxy)phenyl)-1,2,4-oxadiazol-3-yl)benzoate

**DOI:** 10.1021/acsomega.4c06520

**Published:** 2025-01-28

**Authors:** Pooja Mohandas, Abdul Ajees Abdul Salam, Thripthi Nagesh Shenoy, Srinivasulu Maddasani, Santanu Kumar Pal, Channabasaveshwar V. Yelamaggad

**Affiliations:** † Department of Chemistry, Manipal Institute of Technology, Manipal Academy of Higher Education, Manipal 576104, India; ‡ Department of Atomic and Molecular Physics, Manipal Academy of Higher Education, Manipal, Karnataka 576104, India; § Department of Chemical Sciences, Indian Institute of Science Education and Research (IISER) Mohali, Sector-81, Knowledge, Manauli 140306, India; ∥ Centre for Nano and Soft Matter Sciences (CeNS), Arkavathi, Survey No.7, Shivanapura, Dasanapura Hobli, Bengaluru 562162, India; ⊥ SJB Institute of Technology, Health & Education City, Kengeri, Bengaluru 560060, India

## Abstract

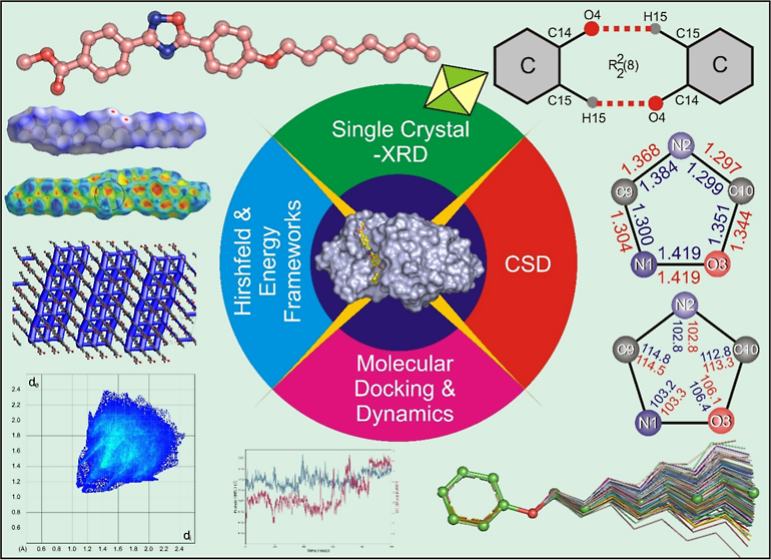

1,2,4-Oxadiazoles are well recognized for their exceptional physical, chemical, and pharmacokinetic properties, making them promising candidates for various therapeutic applications. These include treatments for cystic fibrosis, Duchenne muscular dystrophy, Alzheimer’s disease, and a broad spectrum of other therapeutic interventions such as antituberculosis, anticancer, antibiotic, anti-inflammatory, and anticonvulsant activities. In this study, single crystals of a novel 1,2,4-oxadiazole derivative, methyl-4-(5-(4-(octyloxy)phenyl)-1,2,4-oxadiazol-3-yl)benzoate, were grown by a slow evaporation technique. The structural elucidation was performed using X-ray diffraction analysis, confirming the compound’s crystalline structure in the triclinic system. The analysis revealed a linear conformation with bond lengths closely aligned with Cambridge Structural Database (CSD) averages, signifying high precision in the molecular structure. A detailed CSD study identified nine principal configurations of the phenyl octyloxy moiety, underscoring the structural diversity of the compound. Hirshfeld surface analysis highlighted the predominance of C–H···O and C–H···π interactions, with dispersion energy playing a critical role in stabilizing the crystal lattice. Docking studies against key microbial targets, particularly *E. coli* FabH, demonstrated superior binding energies, suggesting significant antimicrobial potential. The comprehensive suite of structural and computational analyses underscores the potential of the synthesized 1,2,4-oxadiazole derivative, which may be one of the promising candidates for antimicrobial drug development. Future *in vitro*, *in vivo* studies will be supportive in optimizing the derivative for enhanced efficacy and further elucidating its pharmacological mechanisms, paving the way for potential clinical applications. This study not only provides insights into the structural and functional properties of a novel 1,2,4-oxadiazole derivative but also highlights its promising role in antimicrobial drug discovery.

## 1 Introduction

Oxadiazole derivatives are distinctive within the realm of organic compounds owing to their unique molecular structure characterized by a ring containing one oxygen atom and two nitrogen atoms. This molecular configuration confers upon them remarkable polarity and dipole moments, rendering them highly versatile for applications in both medicinal and materials chemistry. Among the various isomeric forms of oxadiazoles, the 1,2,4-oxadiazole isomer stands out due to its diverse array of physical, chemical, and pharmacokinetic properties. These properties underpin its broad spectrum of biological and physiological activities, making it a promising candidate for various therapeutic interventions.^1−10^

1,2,4-Oxadiazoles have shown remarkable efficacy in treating a wide range of medical conditions, including cystic fibrosis, Duchenne muscular dystrophy, Alzheimer’s disease, cancer, and various inflammatory and infectious diseases.^11−16^ These compounds are widely used in pharmaceuticals, with several active pharmaceutical ingredients already on the market.^11−16^ The pharmacophoric properties of 1,2,4-oxadiazoles are also demonstrated by natural products like phidianidine A and B, underscoring their significance in drug development.^16^ A recent review highlights the versatility of 1,2,4-oxadiazole derivatives across various biological activities.^17^ For instance, Loboda et al. synthesized 3,5-substituted 1,2,4-oxadiazoles, identifying compound 64 as a potent DNA topoisomerase IIα inhibitor with unique cellular mechanisms (IC_50_ = 147.7 μM).^18^ Zhang et al. developed anti-inflammatory 1,2,4-oxadiazoles, with compound 65 significantly inhibiting NO production and NF-κB activation (IC_50_ = 12.84 ± 0.21  and 1.35 ± 0.39 μM, respectively).^19^ Gao et al. identified compound 66 as a potent XO inhibitor (IC_50_ = 0.36 μM),^20^ while Chen et al. enhanced antimetastatic properties in nanoliposome formulations.^21^ Choi et al. discovered a compound with hepatoprotective and glucose tolerance-improving effects.^22^ Mohan et al. showcased anticancer potential in prostate and breast cancer cells with compound 69,^23^ and Melo de Oliveira et al. reported antiproliferative activity against lung cancer with compounds 70a and 70b.^24^ Egorova et al. synthesized antiviral 1,2,4-oxadiazoles effective against enteroviruses,^25^ and Sucu et al. developed derivatives with significant anti-GBM activity.^26^ Shi et al. introduced neuroprotective 1,2,4-oxadiazoles, with compound 73 showing promise in stroke models.^27^ In addition, Kumar Kushwaha et al. explored antiviral activity against HIV-1,^28^ and Xie et al. designed compounds that protect vascular endothelial cells from oxidative damage.^29^

Beyond their medicinal applications, oxadiazole derivatives find utility in various technical fields due to their unique chemical structure. The extended substituents at C-3 and C-5 positions endow them with achiral ferroelectric mesomorphism, facilitating their use in fast electro-optic responses, electroluminescence, nonlinear optics, and liquid crystal properties.^30−33^ These derivatives are employed in the development of phosphorescent devices, organic light-emitting diodes, energy materials, metal ion sensors, and gas absorbing/releasing systems, highlighting their versatility in technological applications.^30−33^ The aromatic backbone of oxadiazole derivatives, coupled with polar nitrogen and oxygen atoms in the ring systems, makes them ideal molecular building blocks for self-assembly through hydrogen bonding and π–π stacking interactions. Understanding these molecular interactions offers valuable insights into designing materials with tailored properties and activities. Single-crystal X-ray diffraction (SC-XRD) analysis serves as a powerful tool for elucidating the molecular structures of oxadiazole-based compounds, providing direct correlations between molecular properties and structure.

In this study, we focus on the structural analysis of a novel 1,2,4-oxadiazole derivative, methyl-4-(5-(4-(octyloxy)phenyl)-1,2,4-oxadiazol-3-yl)benzoate. Through careful experimental techniques, including the slow evaporation method, we grew single crystals suitable for X-ray diffraction analysis. Our investigation aims to uncover the structural, energetic, and functional attributes of this compound, shedding light on its potential applications in drug development and materials science. By providing a comprehensive understanding of this compound, we hope to pave the way for future research endeavors aimed at optimizing its efficacy and exploring its pharmacological mechanisms, thus contributing to advancements in both clinical applications and technological innovations.

## 2 Experimental

### 2.1 Synthesis, Characterization, and Crystal Growth

**Scheme 1:**
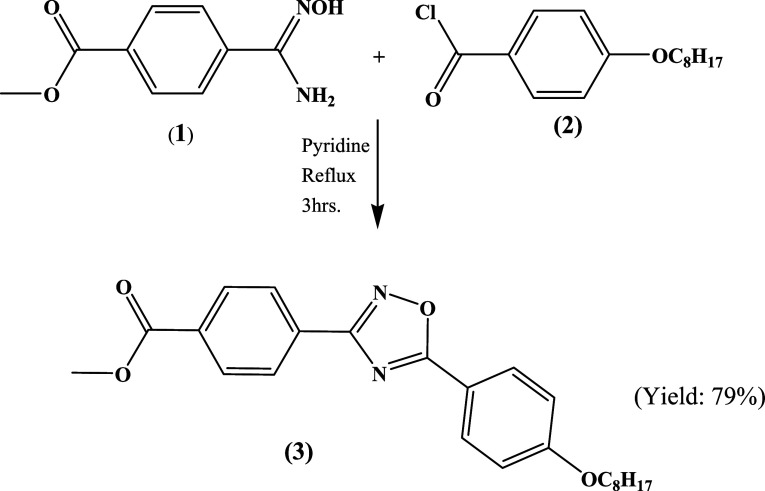
Synthetic Route for the Preparation of 1,2,4-Oxadiazole Derivative

The detailed procedure for the preparation and characterization of methyl-4-(5-(4-(octyloxy)phenyl)-1,2,4-oxadiazol-3-yl)benzoate (3) was reported^34^ elsewhere. 4-Cyano-methylbenzoate was converted into the corresponding amidoxime (1) by treating it with hydroxylamine hydrochloride in a basic medium. 4-Hydroxybenzoic acid was converted into 4-octyloxybenzoic acid which is further transformed into its acid chloride by treating it with thionyl chloride. 4-Octyloxybenzoyl chloride (2) was treated with the amidoxime (1), yielded the final product, viz., 1,2,4-oxadiazole derivative (3). The reaction involved and the molecular structure of the final product are depicted in Scheme 1. The crude product was purified by column chromatography, which yielded shiny crystals after distilling off the solvent. The compound was dissolved in a mixture of dichloromethane and dimethylformamide (1:9) solvents, and its single crystal was grown by the slow evaporation technique at room temperature (25 °C). A colorless crystal measuring 0.30 × 0.25 × 0.20 mm was obtained with a triclinic phase structure.

The obtained crystal was ascertained for its molecular structure by various standard characterization methods which included elemental analyses and various spectroscopic methods, such as UV–visible, ATR-FTIR (Supporting Information Figure S1), ^1^H NMR (Supporting Information Figure S2), ^13^C NMR (Supporting Information Figure S3), and mass spectrometry (ESI-HRMS) (Supporting Information Figure S4), and the relevant data are given in the Supporting Information. The optical textures of the mesophases (Nematic and Smectic A) exhibited by the compound are given as Supporting Information Figure S5, and the differential scanning calorimetry (DSC) thermograms of the same are given as Supporting Information Figure S6.

### 2.2 SC-XRD Data Collection, Structure Determination, and Refinement

SC-XRD data were collected at 298 K on a Rigaku XtaLAB mini diffractometer with a Mercury375/M CCD detector using Mo Kα radiation (λ = 0.71073 Å). The data set was processed using Crystal Clear. Using Olex2,^35^ the structure was solved with the SHELXT^36^ and SHELXL program. All of the non-hydrogen atoms were revealed in the first difference Fourier map itself. All of the hydrogen atoms were positioned geometrically and refined using a riding model. The packing diagrams were generated using MERCURY software.^37^ Crystallographic illustration and molecular graphics were prepared using ORTEP software and the hydrogen bonds are calculated using PARST program.^38^

### 2.3 Cambridge Structural Database (CSD) Studies

The structural similarity, geometry verification, and conformational analysis of the title compound were conducted using CSD version 5.45. This investigation made use of several tools available within the CSD suite. To assess structural similarity, Conquest was utilized to generate molecular representations and identify analogous structures within the extensive CSD database.^39^ Concurrently, Mogul was employed to scrutinize geometric parameters, ensuring compliance with established structural norms.^37^ Additionally, Mercury facilitated detailed conformational analysis, offering insights into molecular flexibility and identifying energetically favorable conformations.^37^ To understand the conformational changes in the octyloxy moiety, a search was conducted in Conquest without any restrictions. The resultant molecules were manually verified and processed individually by using Mercury. PyMol^40^ was employed to superimpose the molecules using the pair-fitting option, and the CSD entries also known as CCDC (Cambridge Crystallographic Data Centre) entries were overlaid on the title compound.

### 2.4 Hirshfeld Surface Calculations

The molecules in a single crystal are held together by noncovalent interactions. The concept of the Hirshfeld surface was developed by a vision to show the space occupied by a molecule in a crystal, giving insight into the individual molecular fragment densities.^41^ This powerful tool gives the virtual visualization of intermolecular close contacts in a crystal by analyzing the size and shape of the Hirshfeld surface; this provides qualitative and quantitative information about intermolecular interactions. The Hirshfeld surface is a 3D representation of a set of points in which the electron density of a molecule is attributed to the electron density of all the other molecules. This is built based on electron distribution by calculating the sum of spherical atom electron densities.^42^ The isosurface is then obtained, and each point on the surface is defined by two distances, *d*_e_ and *d*_i_, representing the distance between the point on the Hirshfeld surface and the closest molecule outside and inside the surface, respectively. The region of intermolecular interactions is mapped by normalized contact distance (*d_norm_*), expressed as *d*_*norm*_ = (*d*_i_ – *r*_i_^vdw^)/ *r*_i_^vdw^ + (*d*_e_ – *r*_e_^vdw^)/*r*_e_^vdw^. The value of *d*_*norm*_ can be positive or negative depending on the intermolecular contacts compared to van der Waals radii (*r*_i_^vdw^). Additionally, the combination of “*d*_e_” and “*d*_i_” in the form of two-dimensional (2D) fingerprint (FP) plots is utilized to provide quantitative information regarding the nature and type of intermolecular contacts in the immediate vicinity of each molecule in the asymmetric unit. 2D FP plots are computed for individual interatomic contacts and overall interactions. The reciprocal contact of each interatomic contact is also considered in the calculation of individual interatomic contacts. Two additional colored properties, based on the local curvature of the surface, such as shape index and curvedness, can be specified. The Hirshfeld surfaces, shape index, curvedness, and 2D FP plots (full and resolved) presented in this paper were generated using Crystal Explorer 21.5.^43^

### 2.5 Ligand Preparation

SMILES notation of the standard antibiotics chloramphenicol and streptomycin and antifungal drug fluconazole were obtained from PubChem (https://pubchem.ncbi.nlm.nih.gov/). The SMILES notation of individual ligands retrieved from PubChem were converted into PDB format using an online server offered by NovoPro Bioscience (https://www.novoprolabs.com/tools/smiles2pdb). The structure of title compound was obtained as a CIF file, which was converted to PDB format using PyMOL (version 2.5.7).^40^ The standard drugs chloramphenicol, streptomycin, and fluconazole and the title compound were prepared and converted into PDBQT format for molecular docking analysis using Open Babel software.^44^

### 2.6 Receptor Preparation

The 3D coordinates of molecular targets from Gram-negative organisms (*Escherichia coli* and *Salmonella typhi*), Gram-positive organisms (*Staphylococcus aureus* and *Streptococcus mutans*), fungus (*Candida albicans*), and protozoan (*Trypanosoma brucei*) were obtained from RCSB Protein Data Bank (https://www.rcsb.org/) to assess the potential antimicrobial activity of the title compound. Eight specific molecular targets from various microorganisms, *E. coli* (PDB id: 1HNJ, 4KFG), *S. typhi* (PDB id: 5E68), *S. aureus* (PDB id: 5ZH8), *S. mutans* (PDB id: 3AIE, 4TQX), and *C. albicans* (PDB id: 4LEB), and *T. brucei* (PDB id: 4MW2) were used for *in silico* analysis. Protein Preparation Wizard of the Schrodinger drug discovery suite (Version 2023-3)^45^ was used for preparation and energy minimization of the molecular targets before docking. The simulation pH was adjusted to 7.4, and all associated cofactors and water molecules were eliminated prior to protein docking. Subsequently, the prepared proteins were saved in the PDB format. Molecular targets with cocrystallized ligands had those ligands removed from their active sites. Alternatively, proteins without cocrystallized ligands were subjected to the SiteMap analysis module of Schrodinger Maestro to identify the potential druggable site.

### 2.7 Molecular Docking

Molecular docking of the standard antimicrobial compounds and the title compound was performed using AutoDock Vina.^46^ The target proteins were prepared and saved in PDBQT files using AutoDock Tools. The grid box dimensions were set with a 1 Å spacing to cover the active site of the target proteins. The grid box dimensions determined for each of the protein targets are mentioned in Supporting Information Table S9. The binding energies were recorded in triplicate for the standard compounds and title compound using different seeds. The molecular interactions were calculated using the Protein–Ligand Interaction Profiler (https://plip-tool.biotec.tu-dresden.de/plip-web/plip/index).^47^ PyMOL molecular visualization software was used to analyze the docked complexes.

### 2.8 MD Simulation and Binding Free Energy Calculations

The Desmond module of the Schrodinger suite (version 2023-3) was used to perform MD simulations of the title compound complexed with molecular targets.^45^ An explicit solvent model with TIP3P confined within an orthorhombic box of 10 Å from the protein surface was used for each complex system. The protein–ligand complexes were subjected to 100 ns simulation under an *NPT* ensemble at 310 K, with trajectory recordings taken every 100 ps. The binding free energy (MM-GBSA) of the protein–ligand complexes was calculated for the trajectories from 10 ns to 100 ns, with a step size of 5 using thermal_mmgbsa.py from Schrodinger Maestro.^45^ Initial 10 ns simulation trajectories were excluded for the system to get equilibrated. Therefore, a total of 180 snapshots from the MD simulations were used for the MM-GBSA calculations.

## 3 Results and Discussion

### 3.1 Structure Solution and Refinement

**Figure 1:**
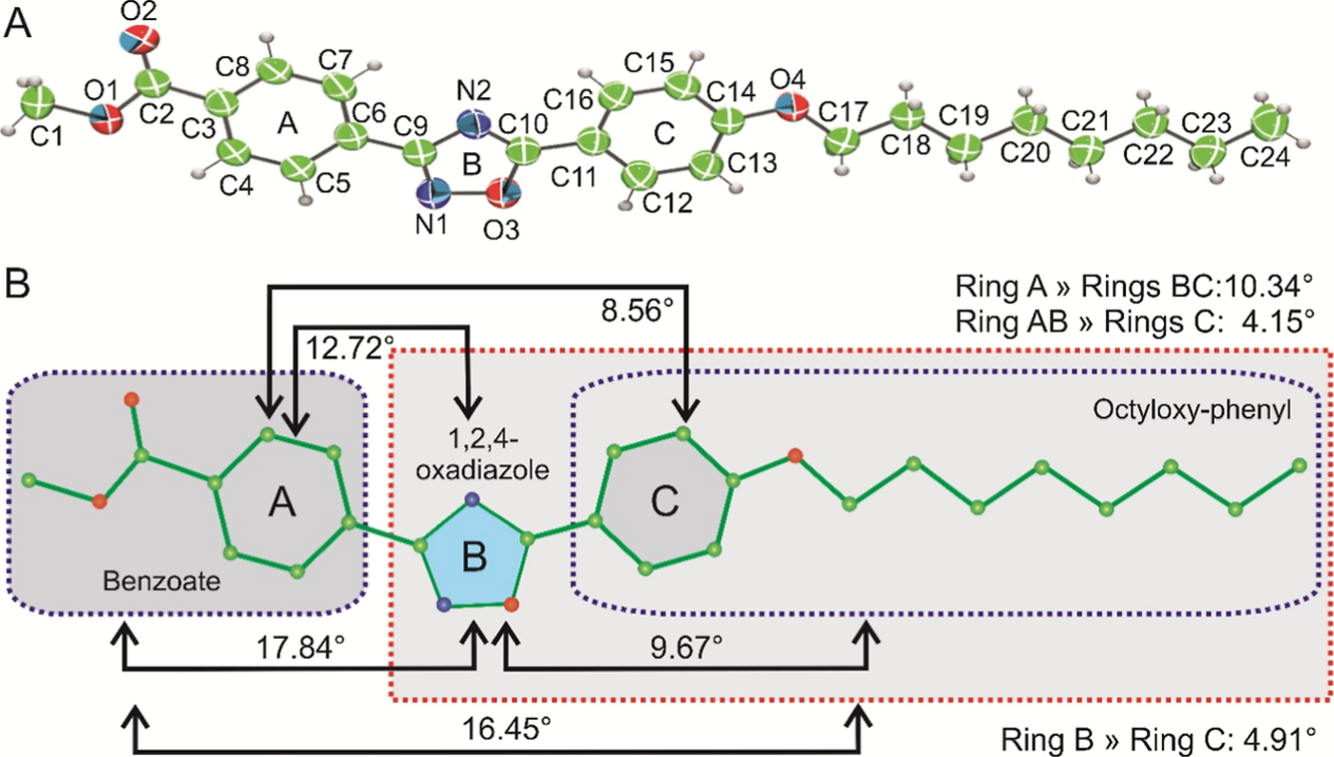
Molecular structure and configuration of the title compound. (A) ORTEP diagram of the molecular structure of the title compound. (B) Depiction of the angles between the planes of various rings, moieties, including their orientations relative to the 1,2,4-oxadiazole, benzoate, and octyloxy–phenyl groups.

The compound crystallizes in the triclinic system with a centrosymmetric P1̅ space group. Cell parameters were refined using least squares in the θ range of 2.581–25.554°, with 4059 reflections collected. Direct methods determined the positions of all non-hydrogen atoms. Further refinement produced a final R-factor of 4.91% and a difference Fourier map with an Δρ_min_ of −0.18 and Δρ_max_ of 0.17 eÅ^–3^. Detailed experimental data are provided in Table 1. Supporting Information Table S1 lists the atomic coordinates and equivalent isotropic factors for the non-hydrogen atoms, while Supporting Information Table S2 provides their anisotropic displacement parameters. Supporting Information Table S3 contains the positional coordinates and isotropic displacement factors for the hydrogen atoms. Figure 1 shows the ORTEP diagram of the molecule with displacement ellipsoids at a 50% probability level.

**Table 1:** Crystal Data and Structure Refinement Details for Methyl-4-(5-(4-(octyloxy)phenyl)-1,2,4-oxadiazol-3-yl)benzoate

formula	C_24_H_28_N_2_O_4_
formula weight	408.501
temperature (K)	298
crystal form, color	block, colorless
crystal system, space group	triclinic, P1̅
a, b, c (Å)	5.9183(2), 8.3874(3), 23.5366(8)
α, β, γ (deg)	92.612(3), 91.533(3), 109.528(4)
volume (Å^3^)	1098.89(7)
Z	2
density (g cm^–3^)	1.235
μ (mm^–1^)	0.084
F(000)	436.3
crystal size (mm)	0.3 × 0.25 × 0.2
radiation	MoKα (λ = 0.71073 Å)
2Θ _min, max,_ (deg)	5.16–51.1
index ranges	–7 ≤ h ≤ 7, −10 ≤ k ≤ 10, −28 ≤ *l* ≤ 28
reflections collected	16313
independent reflections	4057 [*R*_int_ = 0.0313, *R*_σ_ = 0.0234]
data/restraints/parameters	4057/0/273
final R indexes [I ≥ 2σ (I)]	*R*_1_ = 0.0491, w*R*_2_ = 0.1326
final R indexes [all data]	*R*_1_ = 0.0589, w*R*_2_ = 0.1478
Δρ_min_, Δρ_max_ (e Å^–3^)	–0.18, 0.17
GOF on F^2^	1.062
CCDC number	2354206

The bond lengths, bond angles, and torsion angles involving all non-hydrogen atoms are summarized in Supporting Information Tables S4–S6, respectively. The molecule comprises two phenyl rings (labeled A and C, refer to Figure 1A), with average bond lengths of 1.382 and 1.385 Å, respectively, aligning closely with the literature value of 1.380 Å.^48^ Notably, the C5–C6 bond exhibits the highest length at 1.393 Å, while the C15–C16 bond displays the shortest length at 1.373 Å. Similarly, the average bond angles within the phenyl rings measure 120.09°, indicating a minimal deviation.

Moving to the 1,2,4-oxadiazole ring (labeled ring B in Figure 1A), its average bond length is 1.350 Å, while the bond angle measures 108.0°. The N–C bond lengths range from 1.299 to 1.384 Å, with the O–C bond length at 1.351 Å and the highest O–N bond length at 1.419 Å within the ring. Remarkably, there is no deviation observed in the bond angles, aligning well with literature values.^49,50^ Additionally, the octyloxy moiety comprises seven CH_2_ units, with an average bond length of 1.513 Å and an average bond angle of 113.84°. The molecule also contains five C–O bonds and one C=O (C2–O2) moiety, with an average bond length of 1.386 Å and a C2–O2 bond length at 1.201 Å. Notably, the O1–C2 bond exhibits the shortest length at 1.333 Å, while the O1–C1 bond shows the longest length at 1.419 Å, both involving O1 as a common atom within the benzoate moiety.

The torsion angles indicate that the molecule adopts a flat conformation, with torsion angles approaching either zero or 180°. The highest torsion angles recorded are 13.62(17) and 12.56(18)° for O1–C2–C3–C4 and N1–C9–C6–C5, respectively. This observation reveals a slight twist occurring at the bonds C2–C3 and C6–C9, which connect the octyloxy and 1,2,4-oxadiazole moieties to phenyl ring A. The title compound exhibits a linear structure, with an overall length of approximately 26.186 Å. Positioned between the two phenyl rings, the 1,2,4-oxadiazole moiety forms an angle of 159.84° with respect to these rings. All three rings (labeled A, B, and C, as depicted in Figure 1B) are planar, contributing to the overall nearly planar conformation of the molecule. Analysis reveals slight rotations between the planes of these rings: rings B and C exhibit rotations of 12.72 and 8.56°, respectively, in relation to ring A. Additionally, the angle between the planes of rings B and C measures 4.91°. Notably, the CH_3_–CH–C=O moiety displays the significant rotational angle of 13.92° in relation to ring A, while the octyloxy moiety forms an angle of 9.67° with respect to ring C (refer to Figure 1B). In broader perspective, the benzoate moiety exhibits a rotation angle of 16.45° concerning the entire molecule. Similarly, the octyloxy–phenyl moiety displays a rotational angle of 7.32°, concerning the rest of the molecule. Furthermore, concerning the 1,2,4-oxadiazole moiety, the benzoate and octyloxy-phenyl moieties form angles of 17.84 and 9.67°, respectively, as illustrated in Figure 1B.

**Figure 2:**
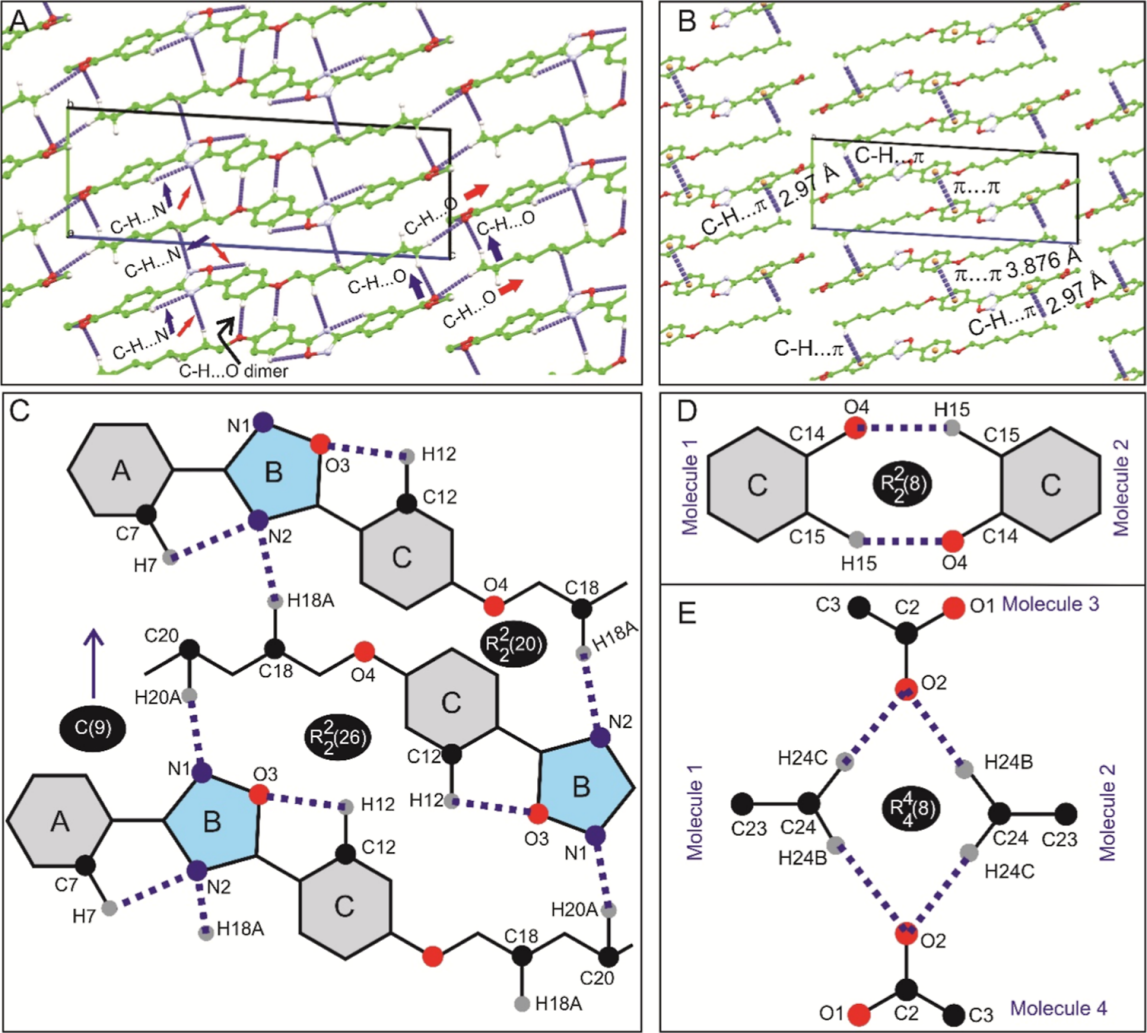
Molecular packing and hydrogen bond network of the title compound. (A) Molecular packing viewed along the *a*-axis, highlighting C–H···O and C–H···N hydrogen bond networks. (B) C–H···π and π···π stacking interactions with marked distances. (C) Infinite chain structure formed by C–H···N hydrogen bond networks along the *ab* plane. (D) C–H···O dimers formed between the phenyl ring. (E) Terminal methyl C–H···O hydrogen bonds forming an infinite network along the *ac* plane. Atoms are shown in a ball-and-stick model; hydrogen bonds are shown in blue dotted lines. Noninteracting hydrogen atoms are omitted for clarity purpose.

Figure 2A illustrates the packing arrangement of the title compound along the *a*-axis. Within the crystal lattice, the molecules are stabilized by both weak intra- and intermolecular hydrogen bonds, as detailed in Table 2. Specifically, two weak intramolecular hydrogen bonds, C_7_–H_7_···N_2_ and C_12_–H_12_···O_3_, are observed (Table 2). Furthermore, four intermolecular C–H···O (C_1_–H_1_C···O_2_, C_15_–H_14_···O_4_, C_24_–H_24_B···O_2_, and C_24_–H_24_C···O_2_) and two intermolecular C–H···N (C_18_–H_18_A···N_2_, C_20_–H_20_A···N_1_) hydrogen bonds contribute to the stabilization of the molecular structure. Notably, the C_15_–H_14_···O_4_ hydrogen bond facilitates the formation of a dimer by connecting the phenyl rings (ring C, as shown in Figure 1) of two adjacent molecules. Similarly, the C_24_–H_24_C···O_2_ bond forms a dimer by linking the edges of the molecules, involving methyl and carbonyl groups (Figure 2A,E). Intriguingly, interactions such as C–H···π with a distance of 2.97 Å, along with π···π stacking interactions, are also observed, contributing to the formation of a supramolecular pattern (Figure 2B). For a detailed examination of the hydrogen bonding network, 2D pictorial representations of hydrogen bonding were made (Figure 2C–E). In Figure 2C, the C_18_–H_18_A···N_2_ and C_20_–H_20_A···N_1_ hydrogen bond network results in a graph set of C(9) along the *ab* plane. Additionally, ring structures are observed between distinct molecules, forming dimers via C_18_–H_18_A···N_2_ and C_20_–H_20_A···N_1_ interactions, with graph set motifs of R_2_^2^(20) and R_2_^2^(26), respectively, along the *ac* plane. Furthermore, a typical C–H···O hydrogen bond with a graph set motif of R_2_^2^(8) is observed, involving C_14_–O_4_ of one molecule and C_15_–H_15_ of another (Figure 2D). Similarly, the terminal methyl group forms another interesting graph set motif of R_4_^4^(8) type C–H···O hydrogen bonding, involving the terminal C=O group of another molecule (Figure 2E). Overall, a single molecule is connected through eight adjacent molecules, stabilizing the structure and forming a supramolecular hydrogen bond network. It is noteworthy that the oxadiazole moiety plays a significant role in facilitating both inter- and intramolecular hydrogen bonding within the *ab* and *ac* planes (Table 2). In general, the octyloxy moiety tends to fold. However, in this molecule, it adopts a linear configuration primarily due to the extensive hydrogen bonds facilitated by the oxadiazole moiety. Specifically, the oxygen atom of the oxadiazole moiety contributes to intramolecular C–H···O interactions (e.g., C_12_–H_12_···O_3_) in conjunction with the adjacent phenyl ring C. Similarly, the nitrogen atoms of the oxadiazole moiety facilitate C–H···N interactions (C_18_–H_18_A···N_2_ and C_20_–H_20_A···N_1_), particularly in association with the octyloxy moiety.

**Table 2:** Possible Hydrogen Bonds of the Title Compound^a^

D-H···A	d(D–H) Å	d(D–A) Å	d(H–A) Å	D–H···A/°
C_7_–H_7_···N_2_	0.930	2.902(2)	2.576	101.08
C_12_–H_12_···O_3_	0.930	2.825(2)	2.512	99.92
C_15_–H_15_···O_4_^#1^	0.930	3.527(2)	2.620	164.89
C_24_–H_24_C···O_2_^#1^	0.960	3.791(2)	2.931	149.70
C_20_–H_20_A···N_1_^#2^	0.970	3.621(3)	2.851	136.96
C_18_–H_18_A···N_2_^#3^	0.970	3.700(2)	2.934	136.73
C_1_–H_1_C···O_2_^#4^	0.960	3.556(3)	2.688	150.51
C_24_–H_24_B···O_2_^#5^	0.960	3.604(2)	2.649	172.96
C_23_A–H_23_A···Cg	0.970	3.800(2)	2.97	144.83

a Symmetry transformation used to generate equivalent positions: ^#1^-*x* – *y* + 1 – *z* + 1; ^#2^-*x* + 1,-*y* + 2, – *z* + 1; ^#3^-*x* + 1,-*y* + 1, – *z* + 1; ^#4^*x* + 1, + *y*, + *z*; ^#5^*x* – 1, + *y* + 1, + *z* + 1.

### 3.2 CSD Studies

#### 3.2.1 Comparison of Bond Length and Bond Angle with CSD Structures

The CSD serves as an invaluable resource for elucidating the structural characteristics of small molecules, providing a vast repository of experimentally determined crystal structures. Studies utilizing the CSD offer a comprehensive understanding of small molecule properties, including bond lengths, bond angles, torsion angles, and molecular configurations.^51,52^ By analyzing the wealth of data within the CSD, researchers gain insights into the intricate spatial arrangements and interactions within molecular structures. This facilitates the exploration of molecular conformations, investigation of chemical bonding patterns, and elucidation of structural motifs. The CSD search results for bond lengths and bond angles of the title compound, including standard deviation, mean, median, minimum, maximum values, and the number of hits, are detailed in Supporting Information Tables S4 and S5.

**Figure 3:**
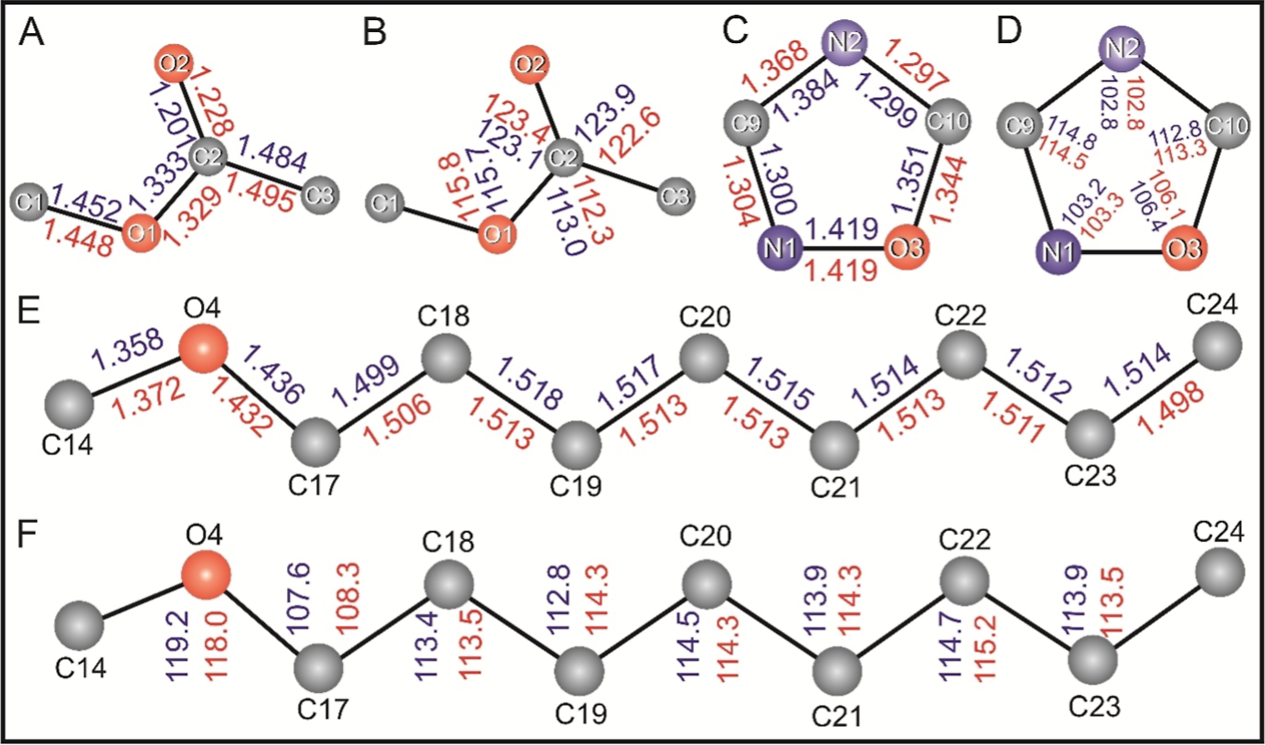
Comparison of bond lengths and bond angles of the title compound with CSD averages. (A) Bond lengths and (B) bond angles of methyl methanoate, (C) bond lengths and (D) bond angles of the 1,2,4-oxadiazole moiety, (E) bond lengths, and (F) bond angles of the octyloxy moieties, compared with CSD average values. The values for the title compound are shown in blue, while the CSD averages are indicated in red.

Thus, the geometry check of the title compound was carried out against the structures deposited in the CSD. The bond length comparison results show that no unusual bond lengths are found in the title compound. The accuracy and precision of the bond length of the title compound compare well with those of CSD searches (Supporting Information Table S4). The standard deviation (σ) and the difference between the title compound and the mean value of CSD entries (Δ) have average values of 0.027 and 0.006 Å, respectively. The highest deviations in σ values are observed for the terminal atoms, with the C_23_–C_24_ CSD average bond length showing an σ value of 0.074 Å. Similarly, the accuracy and precision of valence angle searches were investigated, revealing that the bond angles of the title compound are comparable to the CSD averages. The standard deviation (σ) and the difference (Δ) have average values of 2.343 and 0.82°, respectively (Supporting Information Table S5). The highest σ value of 8.76° is observed for the C21–C22–C23 bond angle. Figure 3 provides a close-up view of the bond length (Figure 3A) and bond angle (Figure 3B) values of the methyl methanoate, oxadiazole (Figure 3C,D), and octyloxy (Figure 3E,F), alongside the CSD average values, highlighting the high degree of similarity between the two sets of values.

#### 3.2.2 Conformational Analysis of Octyloxy Moiety

The octyloxy moiety is known to adopt various configurations due to the flexibility of its methylene groups. To explore the configurations of the phenyl octyloxy moiety, a search of the CSD was conducted using Conquest without any restrictions. This search yielded 263 structures. All 263 structures were manually analyzed by using Mercury, resulting in the extraction of 417 individual molecules containing the phenyl octyloxy moiety. Certain CCDC refcodes, including FIVWIT, JANCVI, RINMEI, TANGOH, TEMSIR, VESWOH, and WALJOM, were excluded from the analysis due to disordered atoms in the phenyl octyloxy moiety. The CCDC refcode NIKKAW contained eight copies of the phenyl octyloxy molecule, while JOJYUF and SEFDOZ each had five copies. Additionally, several CCDC refcodes such as ARAYAT, CUCNUL, HOJVOU, JAYKAW, JESZIR, KAGVIB, KAPCIO, KEDHEJ, NOCSUU, OVOFOX, OVOFUD, QEDDOV, QORCUX, REHLIZ, UCIVOU, UWACEB, UWACIF, UWACOL, UWACUR, and WIRXOK01 each had four phenyl octyloxy molecules. The CCDC refcodes AQEFOU, BUHRUU, GEQWAE, JOKBAP, NAFGAF, SOKTIY, and TAZHUZ contained three molecules each, and 73 other molecules had two phenyl octyloxy molecules.

**Figure 4:**
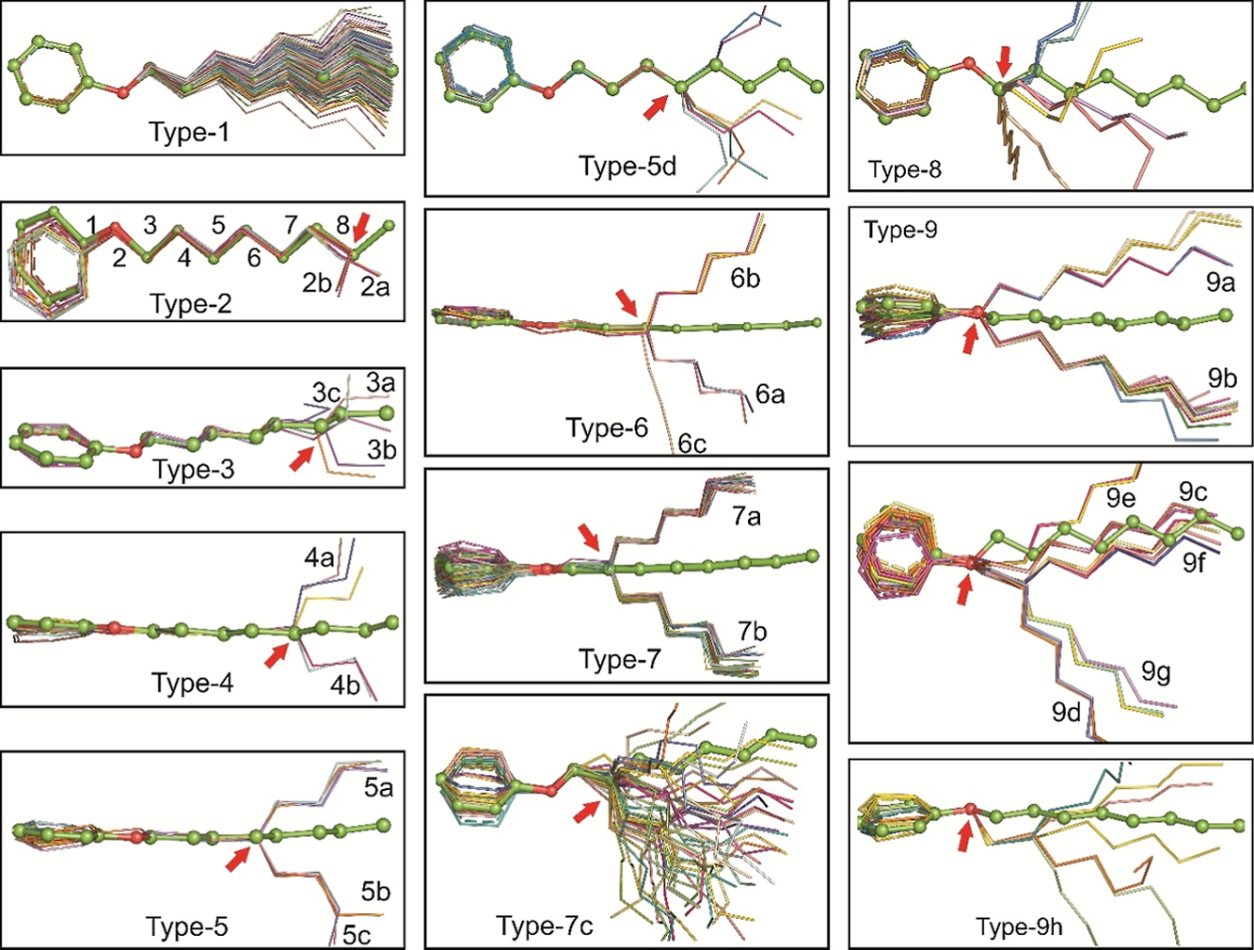
Conformational analysis of the octyloxy moiety. The octyloxy moieties retrieved from the CSD are compared with the title molecule, resulting in the prediction of nine distinct conformation types. These nine predicted types, along with the title compound, are depicted in the figure. Subtypes within each configuration are denoted as a, b, etc. The title compound is represented using a ball-and-stick model, while the CSD structures are illustrated in a line model. Red arrows indicate the specific dihedral angles where twists occur, marked by ± SC configurations.

The analysis of phenyl octyloxy molecules identified nine distinct configuration types, each exhibiting varying degrees of prevalence and diversity. The superposition of phenyl octyloxy molecules with the title compound revealed nine main configuration types, which were further divided into several subtypes, totaling 27 distinct configurations. The dihedral angle deviations for all configurations are detailed in Supporting Information Table S7, with representative figures provided in Figure 4. Type 1 emerged as the most common configuration, encompassing 226 molecules, suggesting that it represents a particularly stable or frequent arrangement. Notably, the title compound adopts the type 1 configuration and superimposes well with these molecules (Figure 4). In contrast, Type 2 was observed in only seven molecules, divided into two subtypes: 2a with three molecules and 2b with four molecules, highlighting its rarity. Type 2 is similar to type 1, except the last dihedral angle, C_21_–C_22_–C_23_–C_24_, adopts an ± SC (synclinal) configuration rather than an AP (antiperiplanar) configuration. In type 1 and the title compound, all eight dihedrals adopt the AP configuration (∼± 180°) (Figure 4). Type 3, also rare with just six molecules, was divided into three subtypes: 3a and 3b, each with two molecules, and 3c, categorized as miscellaneous with two molecules. Type 4, one of the least common configurations, included five molecules and split into subtypes 4a with three molecules and 4b with two molecules, suggesting minor variations. Type 5 showed moderate frequency with 15 molecules, distributed across four subtypes: 5a with four molecules, 5b and 5c each with two molecules, and 5d, a miscellaneous category with seven molecules, indicating a higher degree of variability. Type 6, similar to type 3 in its rarity, consisted of six molecules and three subtypes: 6a with two molecules, 6b with three molecules, and 6c, miscellaneous, with one molecule (Figure 4).

Type 7 was the second most prevalent configuration, comprising 109 molecules. This type displayed significant diversity, with subtypes 7a containing 30 molecules, 7b with 33 molecules, and a large miscellaneous category, 7c, with 46 molecules, reflecting a broad range of possible arrangements. Type 8 was rare, similar to type 2, with seven molecules and no further subdivision. Type 9 showed moderate prevalence with 38 molecules and considerable complexity, divided into eight subtypes: 9a with five molecules, 9b with 12 molecules, 9c with four molecules, 9d and 9e each with two molecules, 9f with four molecules, 9g with three molecules, and a miscellaneous category, 9h, with six molecules.

Statistical analysis reveals that type 1 and type 7 are the dominant configurations (Figure 4), accounting for 54.2% and 26.1% of the total molecules, respectively, suggesting these are likely the most stable or frequently occurring arrangements. Types 2, 3, 4, 6, and 8, representing 1.7%, 1.4%, 1.2%, 1.4%, and 1.7%, respectively, are much less common, indicating these configurations may be less favorable or occur under specific conditions. Types 5 and 9, with 3.6% and 9.1%, respectively, show significant variability, particularly in their miscellaneous subtypes, suggesting multiple stable configurations within these types.

As we discussed earlier, the stereochemical analysis of phenyl octyloxy molecules reveals that the title compound adopts an AP (trans) configuration for all eight dihedral angles, indicating a highly extended and linear conformation. Similarly, type 1 retains the AP (trans) configuration across all dihedral angles, suggesting a high degree of structural similarity and stability to the title compound (Supporting Information Table S7). Type 2 maintains the AP (trans) configuration for the first seven dihedral angles but transitions to a ± SC configuration at the final dihedral angle, indicating a slight twist at the end of the molecule. Type 3 holds the AP configuration until the penultimate dihedral angle, where it adopts an ± SC configuration, suggesting a twist closer to the end of the molecule. Type 4 maintains the AP configuration until the sixth dihedral angle, where it adopts a ± SC configuration, indicating a twist occurring midmolecule. Type 5 shows a ± SC configuration at the fifth dihedral angle, suggesting a twist earlier in the chain, while type 6 features a ± SC configuration at the fourth dihedral angle, indicating an even earlier twist in the molecule. Type 7 presents a ± SC configuration at the third dihedral angle, showing a twist closer to the center of the molecule. Type 8 deviates from ± SC configurations at both the second and third dihedral angles, indicating a significant deviation from the linear structure early in the molecule. Type 9 adopts a ± SC or ± AC configuration at the first dihedral angle, indicating the earliest twist among all types. This progression from type 1 to type 9 reflects a systematic increase in the molecular flexibility and potential interactions influenced by the position of the ± SC or ± AC configuration.

Hydrogen bonding plays a major role in determining the configurations of octyloxy moieties. A notable observation is that type 1 molecules, which are similar to the title compound, typically do not form intramolecular hydrogen bonds within the octyloxy moiety. In contrast, all other types do form intramolecular hydrogen bonds within the octyloxy moiety. In type 1, the terminal CH_3_ group of the octyloxy moiety (e.g., CCDC refcodes AFIHON, and BEQNEU) acts as a donor (C–H···X, where X represents any atom) and forms hydrogen bonds with adjacent molecules arranged in a parallel orientation. Additionally, one or two of the CH_2_ groups also act as donors (e.g., CCDC refcodes AFUTAZ, BEQNEU) and form C–H···X hydrogen bonds with adjacent molecules oriented perpendicularly. The tendency of other types to form intramolecular hydrogen bonds, either within the octyloxy moiety or with a nearby phenyl ring, often necessitates a bending of the octyloxy moiety to adopt a ± SC configuration instead of the AP configuration. This bending facilitates the formation of these intramolecular hydrogen bonds, contributing to the structural diversity observed among the different types. Understanding these hydrogen bonding interactions provides valuable insight into the stability and behavior of various octyloxy configurations, influencing their potential applications and molecular behavior.

### 3.3 Hirshfeld Surface Analysis

**Figure 5:**
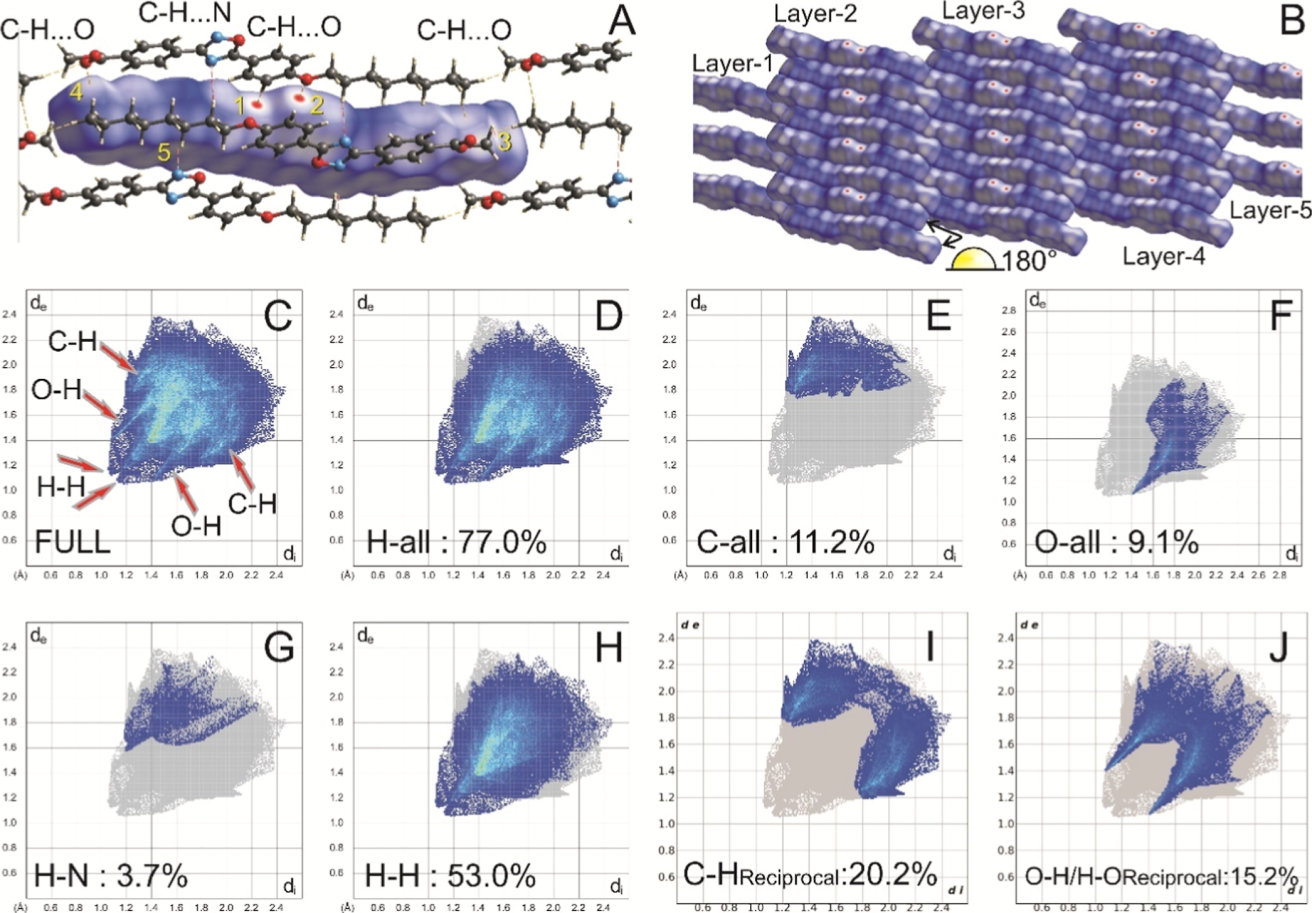
Hirshfeld surface (HS) analysis for the title compound. (A) The HS map for the title compound was generated and mapped with d*_norm_*. Labels 1 and 2 denote the presence of the C–H···O dimer. (B) 3D arrangement of the title compound in different layers. (C) 2D FP plots for the title compound were generated, including full FP (C) and specific pairs of atom types: H-all (D), C-all (E), O-all (F), H–N (G), H–H (H), C–H/H–C (I), and O–H/H–O (J), for the title compound.

The intermolecular interactions of the title compound underwent a comprehensive analysis employing Hirshfeld surface (HS) analysis, complemented by 2D fingerprint (FP) plots for detailed visualization. Illustrated in Figure 5, the HS map and accompanying 2D plots provide insight into the molecular interactions within the compound. Supporting Information Figure S7 showcases the shape index and curvedness of the compound, mapped across specific ranges: *d*_norm_ from −0.0947 to 1.3147 Å, shape index from −0.9954 to 0.9965 Å, and curvedness from ±4.0 Å. The *d*_norm_ surface serves a pivotal role in discerning close intermolecular interactions. Notably, *d*_*norm*_ values can be either negative or positive, indicating shorter or longer intermolecular contacts compared to the van der Waals (vdW) radii, respectively. These values are depicted on the Hirshfeld surface with a color scheme: red regions signify closer contacts with negative *d*_norm_ values, while blue regions denote longer contacts with positive *d*_*norm*_ values. White regions indicate contact distances precisely at the vdW separation, denoted by a *d*_*norm*_ value of zero. The bright red dot on the Hirshfeld surface in the *d*_norm_ mapping highlights significant intermolecular interactions, particularly strong C–H···O bonds. Moreover, the electrostatic potential (MEP) displayed on the molecule’s surface offers valuable insights into its chemical properties (Figure 5B). Red areas signify negatively charged electrostatic potential, indicative of protonation and nucleophilic attack sites, while blue areas represent positively charged electrostatic potential, revealing electrophilic sites. Further analysis of the MEP surfaces reveals electron-rich centers, including carbonyl, alkoxy, and methoxy groups containing oxygen, as well as nitrogen and oxygen atoms within the 1, 2, 4-oxadiazole ring. Conversely, alkyl substitution regions appear in blue, indicating an electrophilic site. Interestingly, the three rings in the molecule exhibit almost neutral electrical charge, depicted in white color. Understanding these electrostatic properties provides insights into secondary interactions within crystal packing as well as the electrophilic and nucleophilic sites involved in molecular interactions.

Within the HS representation, distinct features were observed. Two prominent red spots, labeled 1 and 2, signify the presence of strong C–H···O (C_15_–H_15_···O_4_) hydrogen bonds, crucial for the formation of dimers (Figure 5A). Additionally, two less intense red spots, labeled 3 and 4, correspond to the C_24_–H_24_B···O_2_ and C_24_–H_24_C···O_2_ hydrogen bonds, respectively. Furthermore, the presence of spots indicates the occurrence of weak C–H···N hydrogen bonds (C_20_–H_20_A···N_1_ and C_18_–H_18_A···N_2_), contributing to the overall molecular assembly. The unit cell architecture reveals two molecules positioned approximately 180° apart. Notably, the phenyl ring C of each molecule is oriented nearly centrally, while the benzoate moiety of one molecule faces opposite the octyloxy moiety of the other, with a measured distance of approximately 3.9 Å (Figure 5B). These specific spatial arrangements play a pivotal role in shaping the overall molecular packing and arrangement. Overall, the identified interactions, including both strong and weak hydrogen bonds, along with the spatial orientation of molecular moieties within the unit cell, collectively contribute to the formation of distinct molecular layers, culminating in the intricate 3D arrangement of the molecule.

The complete 2D-FP plot of the title compound is depicted in Figure 5C, providing a comprehensive overview of its intermolecular contacts. Additionally, individual FP plots representing various contact types are illustrated in Figure 5D–J. These plots highlight the specific interactions involved in the molecular assembly of the title compound including H-all (77%, Figure 5D), C-all (11.2%, Figure 5E), O-all (9.1%, Figure 5F), N–H/H–N (3.7%, Figure 5G), H–H (53%, Figure 5H), C–H/H–C (20.2%, Figure 5I), and O–H/H–O (15.2%, Figure 5J). Upon closer examination of the divided fingerprints, it is observed that the highest contribution of contacts received by the title compound is from H–H interactions (53%). Notably, in the FP plots, the O-all and H-all contacts manifest as distinct spikes, indicative of dimer formation within the crystal structure through C–H···O hydrogen bonds between adjacent molecules of the title compound (Figure 2D). Furthermore, the contribution of C–H interactions (20.2%) slightly outweighs that of O–H interactions (15.2%), likely attributed to a combination of weak C–H···O, C–H···π, and π···π interactions. This detailed analysis offers valuable insights into the intermolecular interactions governing the structural arrangement of the title compound, shedding light on its molecular packing and stability.

The shape index and curvedness metrics provided critical insights into the diverse intermolecular interactions experienced by the molecules within the crystal lattice, as shown in Supporting Information Figure S7. The shape index is a highly sensitive tool for detecting subtle variations in surface morphology, distinguishing between concave regions, marked by red triangles, which correspond to atoms from π–stacked molecules above, and convex regions, indicated by blue triangles, which highlight ring atoms within the surface. In our study, the red triangles specifically denote C–H···π interactions, which are integral to the formation of a supramolecular pattern, as evidenced by the C–H···π contact at a distance of 2.97 Å and the observed π···π stacking interactions (Figure 2B). Supporting Information Figure S7A,B further corroborates the presence of these interactions with red and blue triangles circled in dashed lines, pointing to the C–H···π contacts. Additionally, the green flat regions highlighted by blue circles on the curved surface (Supporting Information Figure S7C) correspond to significant π interactions, reinforcing the structural stability of the title compound. The consistency between the shape index and the 2D FP plots enhances the reliability of these findings. The curvedness metric, in turn, delineates regions of low and high curvature, with flat regions indicating extensive π interactions, as previously observed in thiazole-containing compounds in the literature.^53,54^

### 3.4 Energy Framework Analysis

**Figure 6:**
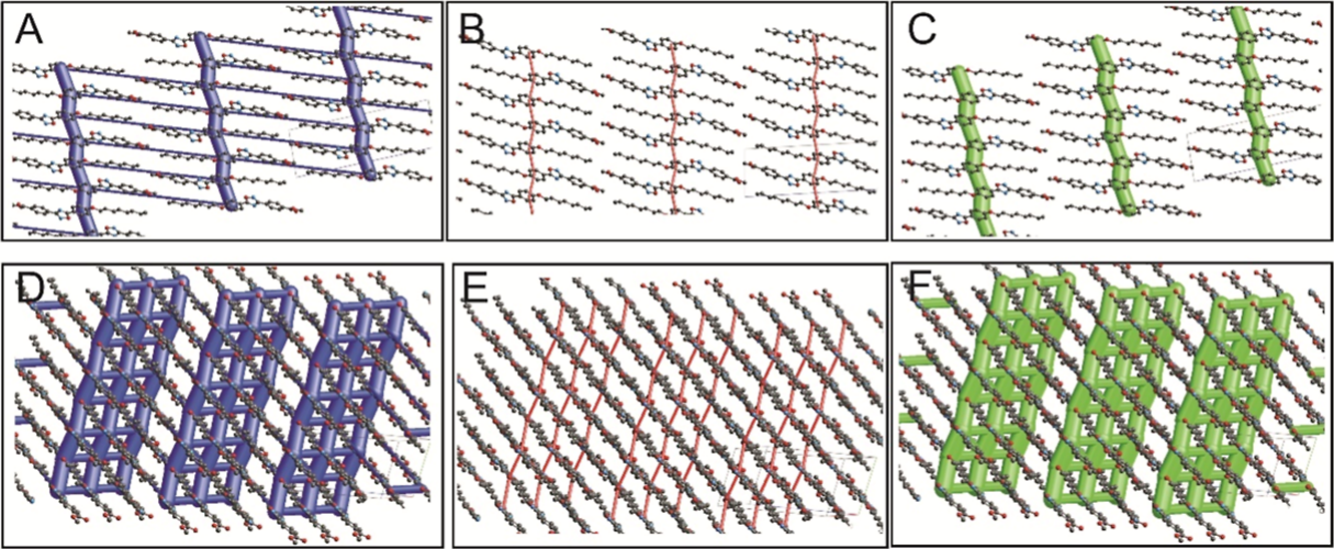
Energy framework analysis of the title compound. The compound is depicted along the *a*-axis (A–C) and slightly reoriented from the ab plane (D–F). The total interaction energy (A,D), electrostatic terms (B,E), and dispersion energy terms (C,F) are depicted in blue, red, and green, respectively. The energy framework interactions were computed using BELYP/G-31G(d,p) electron density functions. The interactions between molecular pairs are represented by cylinders, scaled to a size of 150, connecting the centroids of each pair of molecules. Energies below −10 kJ/mol are omitted for clarity.

Energy framework analysis serves as a crucial tool for comprehending crystal packing dynamics and visualizing interaction topologies rooted in electrostatic, polarization, and exchange-repulsion phenomena. The interaction energies, quantified in kJ/mol, derived from energy framework calculations using Crystal Explorer, are summarized in Supporting Information Table S8. In this analysis, the title compound exhibits a notable dominance of dispersion energy (−108.8 kJ/mol), while electrostatic energies remain relatively low, recorded at −14.1 kJ/mol. It is noteworthy that the total energy profile predominantly mirrors the dispersion energy contribution. Figure 6 presents energy framework interaction plots for the title compound, delineating energy components, such as *E*_tot_, *E*_ele_, and *E*_disp_. The analysis reveals that the title compound forms a parallel network in both the *ab* and *ac* planes, primarily facilitated by the phenyl moiety, with a stronger affinity observed in the *ab* plane. Further examination of the energy framework sheds light on the intricate intermolecular interactions governing the crystal packing of the title compound. The dispersion energy, being the predominant force, suggests significant vdW interactions between the neighboring molecules. Meanwhile, the relatively lower electrostatic energies indicate a lesser contribution from ionic interactions, highlighting the predominantly nonpolar nature of the crystal lattice. This parallel network formation, particularly emphasized in the ab plane, underscores the directional alignment of molecular assemblies indicative of potential structural preferences and packing motifs within the crystal lattice.

### 3.5 Molecular Docking

The data from Antimicrobial Resistance Collaboration has listed *E. coli* as one of the major causes of death due to antimicrobial resistance (AMR).^55^ The antimicrobial efficacy of the title compound was studied using *in silico* docking against *E. coli* FabH and DNA gyrase B enzymes. The enzyme *E. coli* FabH plays a vital role in initiating bacterial fatty acid synthesis and is conserved among Gram-positive and Gram-negative organisms, with no human homologues.^56^ Likewise, *E. coli* DNA gyrase is a well-studied bacterial enzyme with two subunits, contributing to bacterial survival via DNA replication, transcription, and recombination.^57,58^ Targeting DNA gyrase can therefore hinder the crucial cellular processes, causing bacterial cell death. Therefore, *E. coli* FabH and DNA gyrase B are significant targets in combating AMR. The existing crystal structures of FabH (PDB id: 1HNJ) and DNA gyrase B (PDB id: 4KFG) from *E. coli* were retrieved to evaluate the effect of the title compound on these targets. *S. typhi*, the causative agent of typhoid fever, expresses the LuxS quorum sensing gene that is responsible for bacterial virulence and pathogenesis. Targeting this protein interferes with the bacterial protein synthesis and hence regarded as one of the crucial targets to combat Salmonella infections (PDB id: 5E68).^59^

The emergence of methicillin-resistant *S. aureus* strains has left only a few treatments effective. This is due to the overuse of β-lactam antibiotics.^60^*S. aureus* causes bacterial meningitis, nosocomial infections, and skin infections.^61^ Singh and colleagues have reported the association between methicillin resistance among *S. aureus* strains and FmtA. Methicillin resistance is affected upon elimination of *FmtA* gene, and therefore, FmtA is a potential target of *S. aureus* that when targeted might reverse methicillin resistance.^61^ We obtained the available crystal structure of FmtA (PDB id: 5ZH8) from *S. aureus* for examining the antimicrobial potential of the title compound. Dental caries formed by Gram-positive *S. mutans* is a biofilm-associated condition causing AMR faced in dentistry.^62^ Extracellular polymeric substances are a virulence factor responsible for biofilm formation, and its synthesis requires the secretion of glucosyltransferases (GTFs) by the pathogen. Anchoring and aggregation of biofilm on a dental surface requires the assistance of water insoluble glucans. Such water-insoluble glucans are synthesized by Glucosyltransferase-SI (GTF-SI).^63^ Enzymes such as sortase A (SrtA) facilitate covalent attachment of different surface proteins that contribute to virulence on host surfaces. Therefore, GTFs and SrtA promote the formation of bacterial biofilm formation. Targeting SrtA encoding gene decreases the ability of surface proteins to adhere to mucosal surfaces.^64^ As a result, targeting GTFs and SrtA can help in treating and managing dental caries.^63,64^ We employed the existing crystal structures of GTF-SI (PDB id: 3AIE) and SrtA (PDB id: 4TQX) from *S. mutans* to computationally investigate the impact of the title compound on these targets.

Bloodstream-associated nosocomial infections in humans are mainly caused by *C. albicans*. The Als glycoprotein family found in *Candida sp*. facilitates fungal attachment to host cells, thereby promoting the invasion and formation of biofilms. When the ALS gene is removed, *C. albicans* shows decreased adhesion to host cells.^65^ By targeting Als glycoprotein (PDB id: 4LEB) of *C. albicans*, it may be possible to hinder the attachment of fungal species to host cells and consequently prevent biofilm formation. Moreover, the study also utilized tRNA synthetase enzyme (PDB id: 4MW2) as a target for computational analysis.

Molecular docking in drug discovery is a computational approach to predict the optimal protein–ligand interaction and binding energy effectively.^66,67^ In the current study, molecular docking and dynamics were performed to analyze the binding efficiency of the title compound against different molecular targets of AMR causing Gram-negative and Gram-positive bacteria, a fungus, and a protozoan. For our study, a total of eight molecular targets from two Gram-negative organisms, *E. coli* and *S. typhi*, two Gram-positive organisms, *S. aureus* and *S. mutans,* a fungus, *C. albicans*, were used to study the antimicrobial potential of the title compound. Additionally, tRNA synthetase from *T. brucei* was also included in our study. Chloramphenicol, streptomycin, and fluconazole were used as standard antimicrobial drugs against Gram-negative and Gram-positive bacteria, and *C. albicans*, respectively. Fluconazole was also used as a standard drug for *T. brucei*.

The binding energies of the standard drugs and title compound along with the MM-GBSA energies for the title compound are summarized in Table 3. The molecular targets from *E. coli* used in our study are FabH (PDB id: 1HNJ) and DNA gyrase B (PDB id: 4KFG). Among the two targets, the binding energy of the standard drug chloramphenicol was −6.1 kcal/mol, while the title compound showed a binding energy of −7.3 kcal/mol with FabH (Table 3). Within the catalytic site of DNA gyrase B, the binding energy of the standard drug chloramphenicol (−7.9 kcal/mol) was significant compared to the title compound (−3.4 kcal/mol). Similarly, chloramphenicol showed better binding energy (−7.3 kcal/mol) than the title compound (−6.6 kcal/mol) in the active site of the LuxS quorum sensor protein of *S. typhi*. Therefore, based on the docking scores, the complex of the title compound with DNA gyrase B and LuxS quorum sensor protein were omitted from further study. Evaluation of binding energy of the title compound and standard drug streptomycin among FmtA (PDB 5ZH8) of Gram-positive *S. aureus* showed that the title compound showed a binding energy of −6.0 kcal/mol, while streptomycin showed a binding energy of −5.3 kcal/mol. This shows the significant binding of the title compound in the active site of FmtA compared to streptomycin. GTF-SI (PDB id: 3AIE) and SrtA (PDB id: 4TQX) were the studied molecular targets from *S. mutans*. Compared to the two targets, the standard drug streptomycin exerted a better binding energy with GTF-SI (−7.5 kcal/mol) than with SrtA (−5.2 kcal/mol). Likewise, the title compound exerted a better binding energy with GTF-SI (−8.1 kcal/mol) than with SrtA (−6.1 kcal/mol). Overall, the title compound exerted better binding energies in both molecular targets compared to the docked standard drug streptomycin in these targets. For Als-3 adhesion protein from *C. albicans* (PDB id: 4LEB) and methionyl tRNA synthetase from *T. brucei* (PDB id: 4MW2), fluconazole was considered as a standard drug. Within the Als3 adhesion protein catalytic site, fluconazole showed a binding energy of −6.4 kcal/mol, whereas the title compound showed a superior binding energy of −7.3 kcal/mol. Within the methionyl tRNA synthetase active site also, the title compound (−6.7 kcal/mol) showed superior binding energy than fluconazole (−6.1 kcal/mol). Overall, the title compound showed superior binding energy compared to the respective standard drugs among six of the eight studied molecular targets *E. coli* FabH, *S. aureus* FmtA, *S. mutans* GTF-SI and SrtA, *C. albicans* Als-3 adhesion protein, and *T. brucei* methionyl tRNA synthetase (Table 3).

**Table 3:** Binding Energies (BE; kcal/mol) and Molecular Interactions of the Title Compound (M) and Standard Drugs—Chloramphenicol (C), Streptomycin (S), and Fluconazole (F)—within the Active Sites of Six Targeted Proteins from Gram-Positive and Gram-Negative Bacteria, *Fungi*, and *Protozoa*^a^

organism	PDB id	std	BE	H-bonds	HP/π-S/C- π/SB	MM-GBSA
*E. coli*	1HNJ	C	–6.1	Arg36, Gly152, Asn247	Phe213 (HP), Ile250 (HP)	
		M	–7.3	Arg36, Asn247, Asn274	Trp32 (HP), Arg36 (HP), Ile155 (HP), Ile156 (HP), Val212 (HP), Ala246 (HP), Ile250 (HP), His244 (SB)	–76.16
*S. aureus*	5ZH8	S	–5.3	Lys179, Tyr211, Asp213, Arg341, Asn343, Gly345, Gly369, Asn370, Asn371	Lys179 (SB)	
		M	–6.0	Gly345, Phe347, Asn371, Glu372	Ala265 (HP), Leu274 (HP), Phe346 (HP), Val350 (HP), Lys368 (HP)	–49.51
*S. mutans*	3AIE	S	–7.5	Ala478, Glu515, Trp517, His587, Gln592, Asp593	Tyr916 (C-π), Asp477 (SB), Asp588 (SB), Asp909 (SB)	
		M	–8.1	Asn481, Gln592	Leu433 (HP), Asp480 (HP), Trp517 (HP), Tyr916 (HP)	–68.89
	4TQX	S	–5.2	Asn113, His140, Phe142, Asp207, Gly209		
		M	–6.1	Cys205	Ala139 (HP), Val183 (HP), Val190 (HP), Ile191 (HP), Arg213 (HP), Ile215 (HP)	–52.54
*C. albicans*	4LEB	F	–6.4	Tyr21, Ser170, Tyr271	Ala19 (HP), Tyr21 (HP)	
		M	–7.3	Tyr23, Asp169	Arg171 (HP), Leu293 (HP), Trp245 (HP), Lys59 (SB)	–50.39
*T. brucei*	4MW2	F	–6.1	Trp62, Arg112	Asn59 (HP), Ile98 (HP), Ala107 (HP), Trp62 (π-S), Trp108 (π-S)	
		M	–6.7	Asn59, Trp63	Asn59 (HP), Trp62 (HP), Trp63 (HP), Leu75 (HP), Ala107 (HP), Val109 (HP), Trp62 (π-S), Arg73 (SB)	–53.55

a Non-covalent interactions, including hydrophobic interactions (HP), π-stacking (π–S), cation–π (C–π), and salt bridges (SB), are reported. Thermal MM-GBSA calculations (kcal/mol) post-MD simulation for the title compound with the six protein targets are also included.

**Figure 7:**
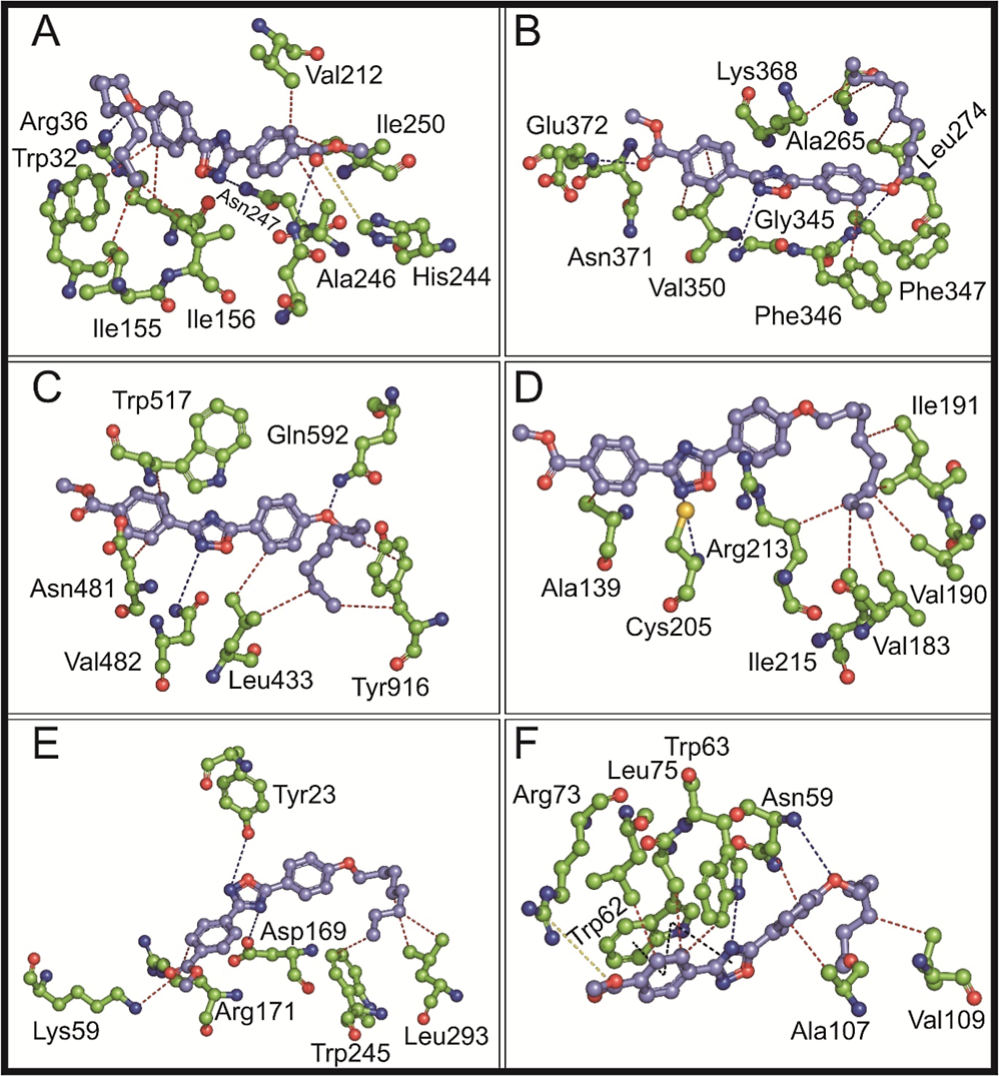
Molecular interactions of the title compound with different microbial targets. Molecular interaction of the title compound with (A) *E. coli* FabH (PDB id: 1HNJ), (B) *S. aureus* FmtA (PDB id: 5ZH8), (C) *S. mutans* glucosyltransferase (PDB id: 3AIE), (D) *S. mutans* sortase A (PDB id: 4TQX), (E) *C. albicans* Als3 adhesion protein (PDB id: 4LEB), and (F) *T. brucei* methionyl tRNA synthetase (PDB id: 4MW2) are shown in ball-and-stick representation. The carbon is shown in lavender for the title compound and green for amino acids. The red- and blue-colored atoms in all molecules indicate oxygen and nitrogen atoms, respectively. H-bonds are represented by blue dotted lines, and red dotted lines represent hydrophobic interactions. π–π interactions and slat bridge interactions are shown in black and green dotted lines, respectively.

The molecular interactions (H-bonds, hydrophobic, π-stack, and salt bridge interactions) of the title compound with the six protein targets were determined (Table 3) and are summarized in Figure 7. Within the *E. coli* FabH enzyme, standard drug chloramphenicol showed H-bonds via Arg36, Gly152, and Asn247 whereas Phe213 and Ile250 participated in hydrophobic interactions (Table 3). The title compound was involved in H-bond formation with Arg36, Asn247, and Asn274. Hydrophobic interactions were made with Trp32, Arg36, Ile155, Ile156, Val212, Ala246, and Ile 250. The compound also participated in salt bridge formation with His244 (Table 3 and Figure 7A). Among chloramphenicol and the title compound, both ligands showed interaction with common residues. For example, both chloramphenicol and the title compound participated in H-bond formation via Arg36 and Asn247 and hydrophobic interactions via Ile250. Moreover, Arg36 also contributed to the hydrophobic interaction with the title compound. Recently, Thongolla et al. reported the antimicrobial activity of phenanthrene-linked oxadiazoles with *E. coli* FabH, where Val212, Ala246, and Ile250 were few of the residues involved in hydrophobic interactions.^56^ In our current study, the title compound also formed hydrophobic interactions with these residues. Within the active site of FmtA of *S. aureus*, the standard drug streptomycin was involved in H-bonds (Lys179, Tyr211, Asp213, Arg341, Asn343, Gly345, Gly369, Asn370, and Asn371) and salt bridge interaction (Lys179) (Table 3). The complex of the FmtA-title compound participated in H-bonds and hydrophobic interactions (Table 3 and Figure 7B). Gly345, Phe347, Asn371, and Glu372 were involved in H-bonds, while Ala265, Leu274, Phe346, Val350, and Lys368 contributed toward hydrophobic interactions. Among streptomycin and the title compound, Gly345 and Asn371 for H-bonds were commonly interacting residues. However, streptomycin did not form hydrophobic interactions, and the title compound was not involved in the salt bride interaction. The title compound formed H-bonds and hydrophobic interactions among both the targets from *S. mutans*, GTF-SI and SrtA. Within the GTF-SI catalytic domain, only two H-bonds were formed with the title compound, each by Asn481 and Gln592 and four amino acids Leu433, Asp480, Trp517, and Tyr916 participated in hydrophobic interactions (Figure 7C). Streptomycin participated in H-bonds via Ala478, Glu515, Trp517, His587, Gln592, Asp593, and cation–π through Tyr916 and salt bridge interactions via Asp477, Asp588, and Asp909 (Table 3). Within the catalytic site of SrtA, streptomycin participated only in H-bonds through Asn113, His140, Phe142, Asp207, and Gly209. SrtA-title compound complex formed only one H-bond with Cys205, and Ala139, Val183, Val190, Ile191, Arg213, and Ile215 were involved in hydrophobic interactions (Figure 7D). The compound interacts with two of the key amino acid residues, Cys205 and Arg213, that are reported to contribute significantly to identification and inhibition of the protein,^64^ indicating the ability of the title compound to tightly interact with the SrtA active site. The complex of standard drug fluconazole with the Als3 adhesion protein of *C. albicans* was involved in H-bonds and hydrophobic interactions. Tyr21, Ser170, and Tyr271 contributed toward the formation of H-bonds, while Ala19 and Tyr21 formed hydrophobic interactions. The Als3-title compound complex formed H-bonds, hydrophobic interactions, and involved a salt bridge formation. H-bonds are formed by Tyr23 and Asp169, while Arg171, Leu293, and Trp245 participate in hydrophobic interactions. Moreover, Lys59 formed a salt bridge (Table 3 and Figure 7E). In the catalytic domain of trypanosomal methionyl tRNA synthetase, the standard drug fluconazole was involved in H-bonds (Trp62 and Arg112), hydrophobic interactions (Asn59, Ile98, and Ala107), and π-stack interactions (Trp62 and Trp108). Asn59 and Trp63 formed H-bonds with the tRNA synthetase-title compound complex. Asn59, Trp62, Trp63, Leu75, Ala107, and Val109 were involved in hydrophobic interactions (Figure 7F). Therefore, Asn59 and Trp63 contribute to the formation of H-bonds, as well as hydrophobic interactions. Additionally, Trp62 and Arg73 participate in π-stack and salt bridge interactions, respectively. Trp62 also contributes to the hydrophobic interaction and salt bridge formation. Comparing the molecular interactions of fluconazole and the title compound in the active site of methionyl tRNA synthetase, the commonly interacting residues include Trp62, Asn59, and Ala107.

### 3.6 MD Simulation

**Figure 8:**
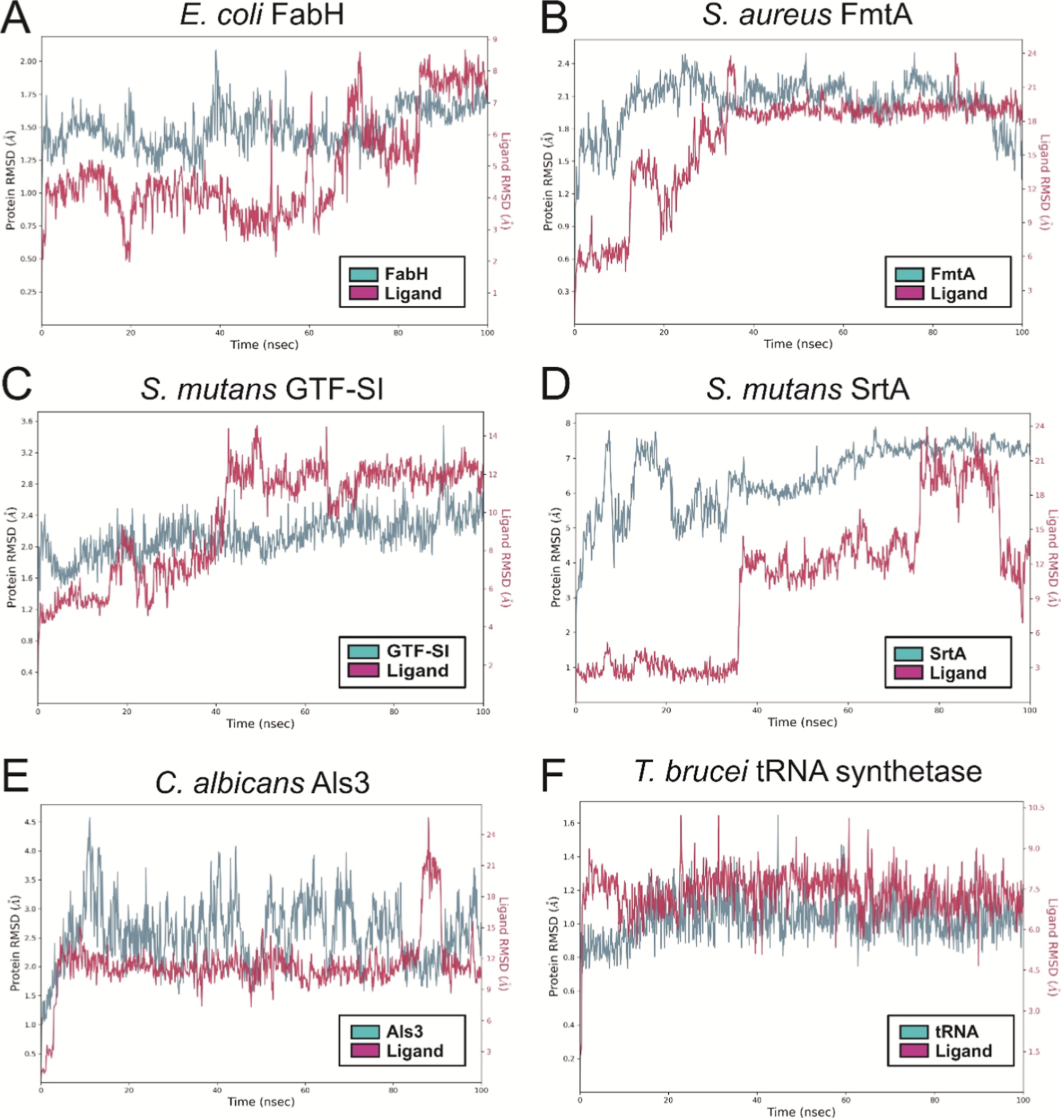
RMSD analysis (in Å) of complex of protein backbone atoms and the title compound during 100 ns MD simulations. Plots represent the RMSD for molecular targets: (A) *E. coli* FabH, (B) *S. aureus* FmtA, (C) *S. mutans* GTF-SI, (D) *S. mutans* SrtA, (E) *C. albicans* Als3, and (F) methionyl tRNA synthetase, each complexed with the synthesized compound. Color codes for the proteins and ligands are provided in the box alongside the respective RMSD plots.

The six protein-title compound complexes, demonstrating superior binding energies compared to those of standard antimicrobial agents, were subjected to 100 ns MD simulations. The proteins involved in these simulations include FabH, FmtA, GTF-SI, SrtA, Als3, and tRNA synthetase, all complexed with the title compound. Notably, the title compound remained stable within the catalytic site of the *E. coli* FabH complex throughout the simulation, as illustrated in Supporting Information Video S1. The ligand RMSD plot reveals an initial RMSD stability up to 4 Å until 62 ns (Figure 8A), followed by an increase to 8.5 Å in the later frames. The abrupt RMSD change at 20 ns corresponds to a rotation of the bond connected to the five-membered ring. The simulation trajectory further indicates the flexibility of the octyloxy moiety, which undergoes rotation across different aliphatic carbons during the simulation. After 62 ns, the title compound exhibits slight dissociation from its original position, likely adjusting itself within the catalytic site, which accounts for the elevated ligand RMSD beyond this point (Supporting Information Video S1).

In the active site of FmtA in *S. aureus*, the title compound exhibits a markedly different binding pattern. The ligand dissociates from its initial docked position within the first quarter of the simulation, relocating to a new site where it remains stable for the rest of the duration, though the aliphatic tail of the compound retains some flexibility (Supporting Information Video S2). This shift is reflected in the ligand RMSD, which remains stable yet elevated, ranging from 18 to 24 Å between 40 and 100 ns (Figure 8B). For the *S. mutans* GTF-SI-title compound complex, the binding behavior is particularly unique. The ligand RMSD fluctuates between 4 and 14 Å throughout the simulation (Figure 8C). Initially, the title compound attempts to reorient itself from the docked site, resulting in an increase in RMSD during the first 25 ns (Figure 8C). During this reorientation, the ligand shifts slightly from its docked position, causing the aliphatic chain to unfold (Supporting Information Video S3). This occurs between 42 and 62 ns, where the RMSD varies between 10 and 14 Å (Figure 8C). After reorienting, the title compound stabilizes with reduced flexibility and movement, leading to a more stable RMSD from 75 to 100 ns (Figure 8C and Supporting Information Video S3).

In the *S. mutans* SrtA-title compound complex, the title compound undergoes a dynamic movement from the initial docked site to an intermediate site and then to a third site, before returning to the intermediate position (Supporting Information Video S4). This movement is reflected in the ligand RMSD, which shows distinct stages: it remains around 3 Å from 0 to 38 ns, increases to 9–15 Å between ∼38 and 75 ns, rises further to 15–24 Å between ∼75 and 95 ns, and finally decreases back to 7–15 Å in the last 5 ns (Figure 8D). The simulation trajectory, coupled with the RMSD data, reveals that the title compound stays in the docked site for the first 38 ns, transitions to an intermediate site where it stabilizes for the next 37 ns, and then reverts to the intermediate site for the final 5 ns of the simulation (Supporting Information Video S4). Throughout this process, the title compound exhibits bond flexibility, in both its aliphatic tail and aromatic core, offering insights into its interaction with the *S. mutans* SrtA enzyme.

In contrast, the title compound forms a relatively stable complex with the Als3 adhesion protein of *C. albicans*. The RMSD of the title compound fluctuates between 9 and 15 Å from 5 to 100 ns, with a notable spike to 24 Å in the final 20 ns (Figure 8E). The initial RMSD fluctuation within the first 5 ns corresponds to a slight reorientation of the compound within the protein’s active site, causing a minor displacement from the docked position. The simulation trajectory highlights the flexibility of the ligand’s aliphatic chain. The significant fluctuation observed between 80 and 100 ns is attributed to the compound transitioning away from and then returning to the active site. Overall, the title compound demonstrates strong interactions with the Als3 protein of *C. albicans* (Supporting Information Video S5). In the active site of methionyl tRNA synthetase, the title compound remains consistently stable throughout the simulation. The aliphatic chain anchors the compound firmly within the active site, while flexibility is primarily seen in the bond between the five-membered ring, the phenyl ring, and the terminal –COOH group of the title compound (Supporting Information Video S6). The RMSD plot shows that the title compound’s RMSD ranges between 6 and 9 Å, with occasional higher fluctuations at 20, 30, 40, 60, and 90 ns, which are attributed to the rotation of bonds in the aliphatic chain (Figure 8F).

As discussed previously, the octyloxy moiety is highly flexible, allowing it to adopt various conformations. During the MD simulations, this flexibility led to significant fluctuations in the octyloxy moiety, resulting in the adoption of distinct conformations across different complexes (Supporting Information Videos S1, S2, S3, S4, S5, and S6). Specifically, complexes with PDB IDs 1HNJ, 5ZH8, 3AIE, and 4LEB adopted type 9 configurations, while those with PDB IDs 4TQX and 4MW2 adopted type 6 and type 8 configurations, respectively. Although these movements caused RMSD fluctuations, as observed in the *E. coli* FabH system (Figure 8A), they also facilitated the formation of several hydrogen bonds (Figure 7).

### 3.7 Binding Free Energy Calculations

Following MD simulation, the six proteins with the title compound complexes were subjected to thermal MM-GBSA calculations, and the binding energies are listed in Table 3. Except for the first 10 ns, trajectory snapshots for the remaining 90 ns were considered for MM-GBSA calculations, with a step size of 5 frames. The binding energy for the *E. coli* FabH-title compound is −76.16 kcal/mol. The binding energy between *S. aureus* FmtA and the title compound was obtained as −49.51 kcal/mol. The complexes of GTF-SI-title compound and SrtA-title compound showed binding energies of −68.89 and −52.54 kcal/mol, respectively. The Als3 adhesion protein-title compound from *C. albicans* exerted a binding energy of −50.39 kcal/mol, whereas the methionyl tRNA synthetase-title compound complex showed a binding energy of −53.55 kcal/mol. Therefore, the title compound complexed with *E. coli* FabH showed a superior binding energy of −76.16 kcal/mol than other protein-title compound complexes.

AMR is one of the global concerns causing an increase in infections especially caused by pan drug and multidrug resistance bacteria, making it difficult to treat the clinically important pathogens with current antibiotics.^68^ One of the concerns against Gram-negative bacteria regarding the antimicrobials is the presence of an outer membrane that restricts most of the bioactive compounds from reaching the intracellular targets. Although fatty acids are present in the membranes of all cells, bacteria have a different process to synthesize them compared to eukaryotes, making it an important target for combating AMR. *E. coli* is considered a high-priority pathogen by WHO.^69^ Consequently, ongoing research remains dedicated to developing or discovering new antimicrobial agents. As previously mentioned, *E. coli* FabH initiates bacterial fatty acid synthesis and is conserved among Gram-positive and Gram-negative organisms, with no human homologues.^56^ Therefore, *E. coli* FabH is one of the extensively studied microbial targets in combating AMR. In our study, we have computationally identified the potential of the title compound in targeting the *E. coli* FabH and other enzymes. To support the computational studies, the title compound should be further evaluated for its antimicrobial activity through *in vitro* assays.

## 4 Conclusions

This study focused on the synthesis and detailed characterization of a novel 1,2,4-oxadiazole derivative, methyl-4-(5-(4-(octyloxy)phenyl)-1,2,4-oxadiazol-3-yl)benzoate. The compound was successfully crystallized using the slow evaporation technique, and its structure was confirmed via X-ray diffraction analysis, which revealed a triclinic crystal system with space group P1̅ and a final R-factor of 4.91%. The molecule adopts a predominantly planar conformation, with maximum torsion angles recorded at 13.62(17)° for O1–C2–C3–C4 and 12.56(18)° for N1–C9–C6–C5. The linear structure of the compound, approximately 26.186 Å in length, is characterized by the 1,2,4-oxadiazole moiety positioned between two phenyl rings, forming an angle of 159.84° relative to these rings.

The stability of the crystal lattice is primarily driven by a network of weak intra- and intermolecular C–H···O and C–H···N hydrogen bonds, along with π···π stacking interactions facilitated by the oxadiazole moiety. A comparative analysis with the CSD revealed no anomalous bond lengths with minor deviations noted mainly in terminal atoms and specific bond angles. Additionally, a detailed investigation of the phenyl octyloxy moiety revealed nine primary configuration types, further subdivided into several subtypes, highlighting the structural diversity and prevalence of these configurations. Notably, type 1 and type 7 configurations were the most common, likely due to their inherent stability. Hydrogen bonding emerged as a crucial determinant of the octyloxy moiety’s configuration, with type 1 configurations notably lacking intramolecular bonds, in contrast to other types.

Hirshfeld surface analysis elucidated the prominence of H–H interactions, with FP plots indicating dimer formation through C–H···O hydrogen bonds. Energy framework analysis further highlighted dispersion energy (−108.8 kJ/mol) as the dominant force contributing to the compound’s stability and packing. The antimicrobial potential of the compound was evaluated through *in silico* docking studies against multiple microbial targets, including *E. coli* FabH and DNA gyrase B enzymes, *S. aureus* FmtA, *C. albicans* Als-3 adhesion protein, and *T. brucei* methionyl tRNA synthetase. Molecular dynamics simulations over 100 ns confirmed the compound’s stability within the catalytic sites of all six targets. Thermal MM-GBSA calculations revealed a notably strong binding energy (−76.16 kcal/mol) for the *E. coli* FabH-title compound complex.

These findings provide a comprehensive understanding of the structural, energetic, and functional properties of this novel 1,2,4-oxadiazole derivative, underscoring its potential as a candidate for antimicrobial drug development. Future research should focus on synthesizing derivatives to enhance antimicrobial efficacy and further investigate the compound’s pharmacological mechanisms, facilitating its progression toward clinical applications and pharmaceutical development.

## Supporting Information Available

Characterization data of methyl-4-(5-(4-(octyloxy)phenyl)-1,2,4-oxadiazol-3-yl)benzoate; ATR-FTIR spectra of methyl-4-(5-(4-(octyloxy)phenyl)-1,2,4-oxadiazol-3-yl)benzoate; ^1^H NMR spectrum of methyl-4-(5-(4-(octyloxy)phenyl)-1,2,4-oxadiazol-3-yl)benzoate (400 MHz; CDCl_3_); ^13^C NMR spectrum of the methyl-4-(5-(4-(octyloxy)phenyl)-1,2,4-oxadiazol-3-yl)benzoate (100 MHz; CDCl_3_); mass spectrum (ESI-HRMS) of methyl-4-(5-(4-(octyloxy)phenyl)-1,2,4-oxadiazol-3-yl)benzoate and HRMS theoretical and calculated values; polarizing optical microscope images of various mesophases; DSC thermograms of the compound; molecular surface analysis of Hirshfeld, shape index, and curvedness; fractional atomic coordinates (×10^4^) and equivalent isotropic displacement parameters (Å^2^ × 10^3^); anisotropic displacement parameters (Å^2^ × 10^3^); hydrogen atom coordinates (Å × 10^4^) and isotropic displacement parameters (Å^2^ × 10^3^); bond lengths of the title compound with CSD structure comparison; bond angles of the title compound with CSD structure comparison; torsion angles of the title compound; conformational analysis of the phenyl octyloxy moiety of the title compound with CSD structures; interaction energies acquired from the energy framework calculations; and grid box dimensions for the molecular targets selected for molecular docking (PDF)
MD simulation of the title compound with *E. coli* FabH protein (MP4)
MD simulation of the title compound with *S. aureus* FmtA protein (MP4)
MD simulation of the title compound with glucosyl transferase GTF-SI of *S. mutans* (MP4)
MD simulation of the title compound with sortase A (SrtA) of *S. mutans* (MP4)
MD simulation of the title compound with Als3 adhesion protein of *C. albicans* (MP4)
MD simulation of the title compound with methionyl tRNA synthetase of *C. albicans* (MP4)
