# Supplementary material for: Structural, CSD, Molecular Docking, Molecular Dynamics, and Hirshfeld Surface Analysis of a New Mesogen, Methyl-4-(5-(4-(octyloxy)phenyl)-1,2,4-oxadiazol-3-yl)benzoate
Source: ACS Omega. 2025 Jan 28;10(5):4336–52. doi: 10.1021/acsomega.4c06520 (PMC11822514; doi:10.1021/acsomega.4c06520)
Supplement: Supplementary file 1 — ao4c06520_si_001.pdf [file ao4c06520_si_001.pdf]

Supporting information

# Structural, CSD, Molecular Docking, Molecular Dynamics and Hirshfeld Surface Analysis of a New Mesogen, Methyl-4-(5-(4-(octyloxy)phenyl)-1,2,4-oxadiazol-3-yl)benzoate

Pooja Mohandas[a], Abdul Ajees Abdul Salam[b,*], Thripthi Nagesh Shenoy[b], Srinivasulu Maddasani[a,*], Santanu Kumar Pal[c], and Channabasaveshwar V. Yelamaggad[a,d,e*]

[a]*Department of Chemistry, Manipal Institute of Technology, Manipal Academy of Higher Education, Manipal – 576104, India.*

[b]*Department of Atomic and Molecular Physics, Manipal Academy of Higher Education, Manipal – 576104, Karnataka, India.*

[c]*Department of Chemical Sciences, Indian Institute of Science Education and Research (IISER) Mohali, Sector-81, Knowledge City, Manauli 140306, India.*

[d]*Centre for Nano and Soft Matter Sciences (CeNS), Arkavathi, Survey No.7, Shivanapura, Dasanapura Hobli, Bengaluru -562162, India.*

[e]*SJB Institute of Technology, Health & Education City, Kengeri, Bengaluru – 560060, India.*

Corresponding authors *E-mail: abdul.ajees@manipal.edu; s.maddasani@manipal.edu; yelamaggad@cens.res.in*

## Table of contents

1. Data S1. Characterization data of methyl-4-(5-(4-(octyloxy)phenyl)-1,2,4-oxadiazol-3-yl)benzoate.
2. Figure S1. ATR-FTIR spectra of the methyl-4-(5-(4-(octyloxy)phenyl)-1,2,4-oxadiazol-3-yl)benzoate
3. Figure S2. $^{1}$H-NMR spectrum of the methyl-4-(5-(4-(octyloxy)phenyl)-1,2,4-oxadiazol-3-yl)benzoate (400 MHz; $CDCl_3$).
4. Figure S3. $^{13}$C-NMR spectrum of the methyl-4-(5-(4-(octyloxy)phenyl)-1,2,4-oxadiazol-3-yl)benzoate (100 MHz; $CDCl_3$).
5. Figure S4. Mass spectrum (ESI-HRMS) the methyl-4-(5-(4-(octyloxy)phenyl)-1,2,4-oxadiazol-3-yl)benzoate and HRMS theoretical and calculated values.
6. Figure S5. Polarizing optical microscope images of mesophases
7. Figure S6. DSC thermograms of the compound
8. Figure S7. Molecular surface analysis of Hirshfeld, shape index, curvedness.
9. Table S1. Fractional Atomic Coordinates ($\times10^4$) and Equivalent Isotropic Displacement Parameters (Å$^2\times10^3$)
10. Table S2. Anisotropic Displacement Parameters (Å$^2\times10^3$)
11. Table S3. Hydrogen Atom Coordinates (Å$\times10^4$) and Isotropic Displacement Parameters (Å$^2\times10^3$)
12. Table S4. Bond lengths of the title compound with CSD structure comparison

13. Table S5. Bond angles of the title compound with CSD structure comparison
14. Table S6. Torsion angles of the title compound
15. Table S7. Conformational analysis of phenyl octyloxy moiety of the title compound with CSD structures.
16. Table S8. Interaction energies acquired from the energy framework calculations
17. Table S9. Grid box dimensions for the molecular targets selected for molecular docking
18. Supplementary Video S1: MD Simulation of the title compound with *E. coli* FabH protein.
19. Supplementary Video S2: MD Simulation of the title compound with *S. aureus* FmtA protein.
20. Supplementary Video S3: MD Simulation of the title compound with glucosyl ransferase GTF-SI of *S. mutans*.
21. Supplementary Video S4: MD Simulation of the title compound with sortase A (SrtA) of *S. mutans*.
22. Supplementary Video S5: MD Simulation of the title compound with Als3 adhesion protein of *C. albicans*.
23. Supplementary Video S6: MD Simulation of the title compound with methionyl tRNA synthetase of *C. albicans*.

**Data S1. Characterization data of methyl-4-(5-(4-(octyloxy)phenyl)-1,2,4-oxadiazol-3-yl)benzoate**

$R_f$ = 0.71 (20 % EtOAc-Hexanes); white solid; yield: 0.17 g (79 %); ATR-FTIR: $\nu_{max}$ in $cm^{-1}$ 2957, 2939, 2917, 2852, 1718, 1612, 1503, 1274, 1172, 1106, 752, and 720; UV-Vis: $\lambda_{max}$ = 268.0 nm, $\varepsilon = 3.4402\times10^{-4}$ L mol $^{-1}$ $cm^{-1}$; $^1H$ NMR (400 MHz, $CDCl_3$): δ 8.15 (d, *J* = 6.8 Hz, 2H, Ar), 8.08 (d, *J* = 5.6 Hz, 2H, Ar), 8.05 (d, *J* = 6.4 Hz, 2H, Ar), 6.94 (d, *J* = 6.8 Hz, 2H, Ar), 3.95 (t, *J* = 4.4 Hz, 2H, $-OCH_2$), 3.87 (s, 3H, $-OCH_3$), 1.74-1.21 (m, 12H, $6\times CH_2$), 0.82 (t, *J* = 9.2 Hz, 3H, $-CH_3$); $^{13}C$ NMR (100 MHz, $CDCl_3$): δ 174.97, 167.06, 165.45, 161.94, 131.25, 130.25, 129.06, 128.99, 126.43, 115.27, 113.97, 67.35, 51.31, 30.78, 28.31, 28.20, 28.07, 24.96, 21.63 and 13.07. ESI-HRMS: m/z Calcd. for $C_{24}H_{28}N_2O_4$: (M+H) 409.21; Found: 409.21; Anal. calcd. for $C_{24}H_{28}N_2O_4$: C, 70.57; H, 6.91; N, 6.86. Found: C, 70.79; H, 6.61; N, 6.93.

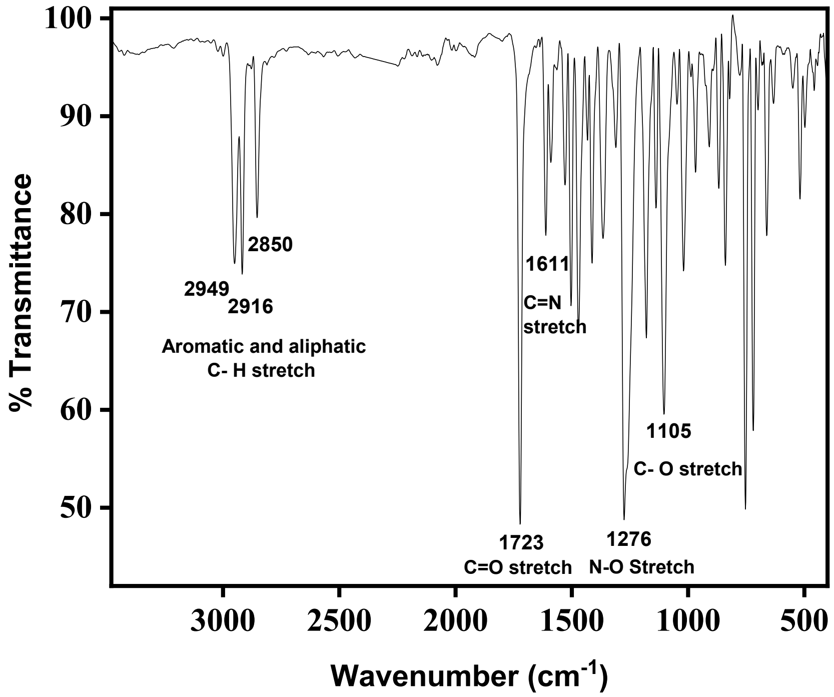


**Figure S1.** ATR-FTIR spectra of the methyl-4-(5-(4-(octyloxy)phenyl)-1,2,4-oxadiazol-3-yl)benzoate

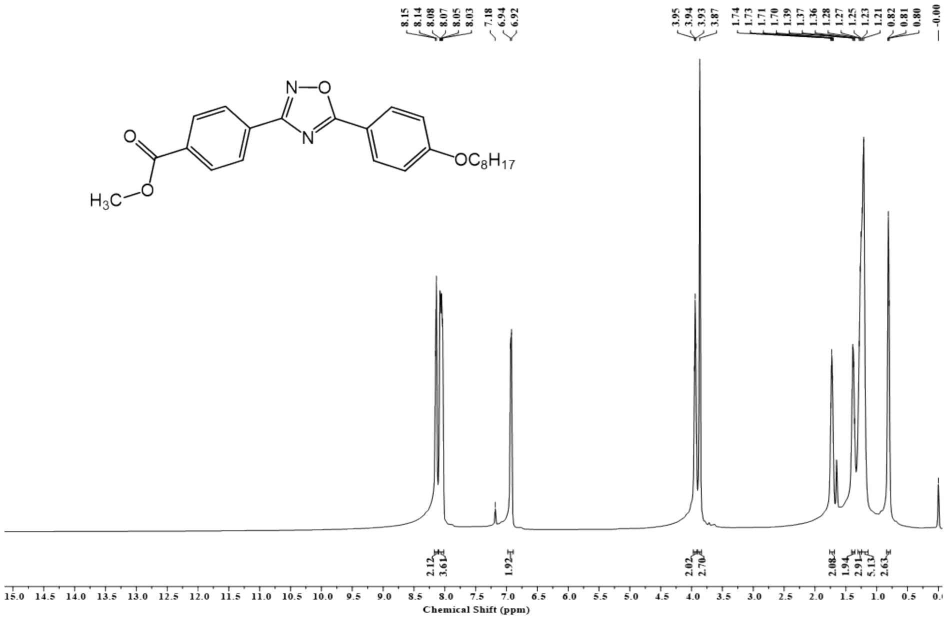

**Figure S2.** $^{1}$H-NMR spectrum of the methyl-4-(5-(4-(octyloxy)phenyl)-1,2,4-oxadiazol-3-yl)benzoate (400 MHz; $CDCl_3$)

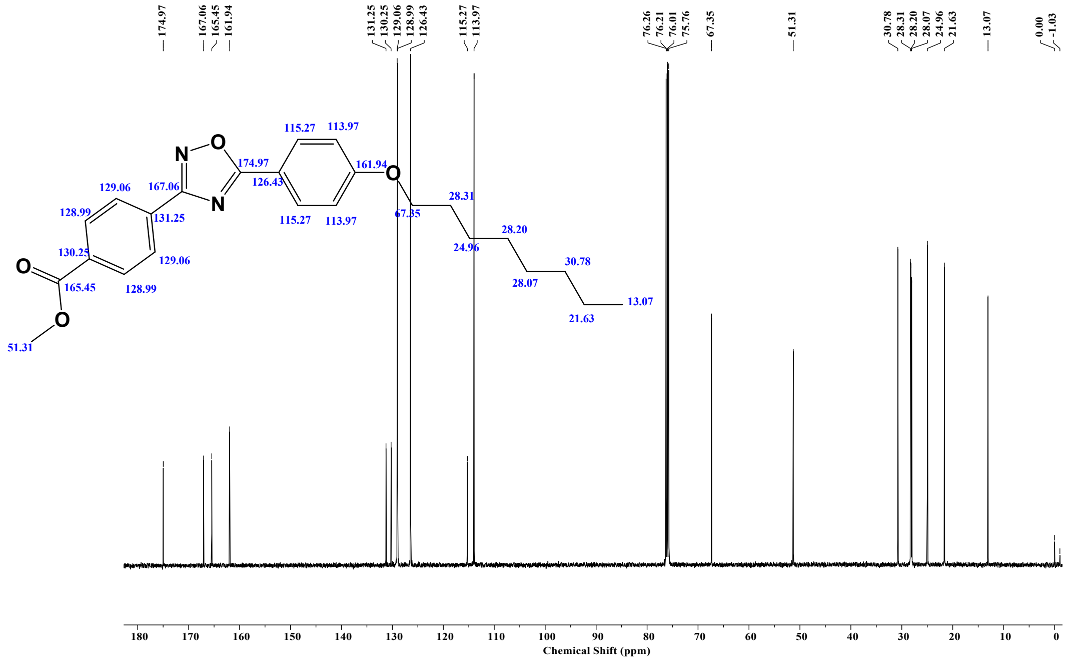

**Figure S3.** $^{13}$C NMR spectrum of the methyl-4-(5-(4-(octyloxy)phenyl)-1,2,4-oxadiazol-3-yl)benzoate(100 MHz; $CDCl_3$)

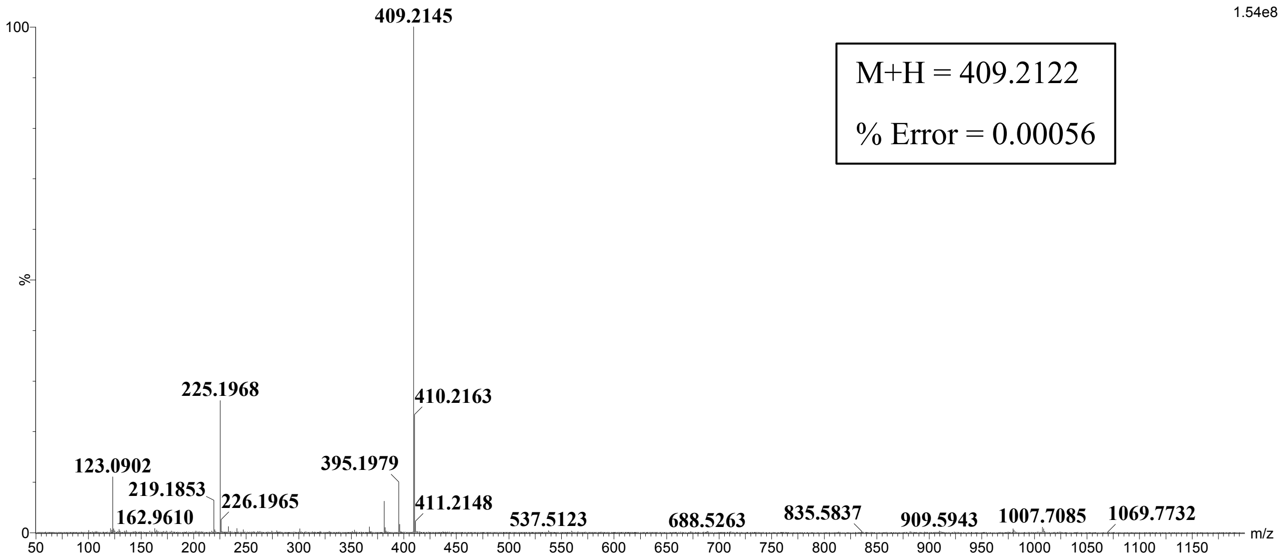


| Theoretical (Exact Mass) | Theoretical (M+H) | Observed Mass |
|---|---|---|
| 408.2049 | 409.2122 | 409.2145 |

**Figure S4.** Mass spectrum (ESI-HRMS) of the methyl-4-(5-(4-(octyloxy)phenyl)-1,2,4-oxadiazol-3-yl)benzoate. The table below the spectrum shows the HRMS calculated and observed values to four decimal places

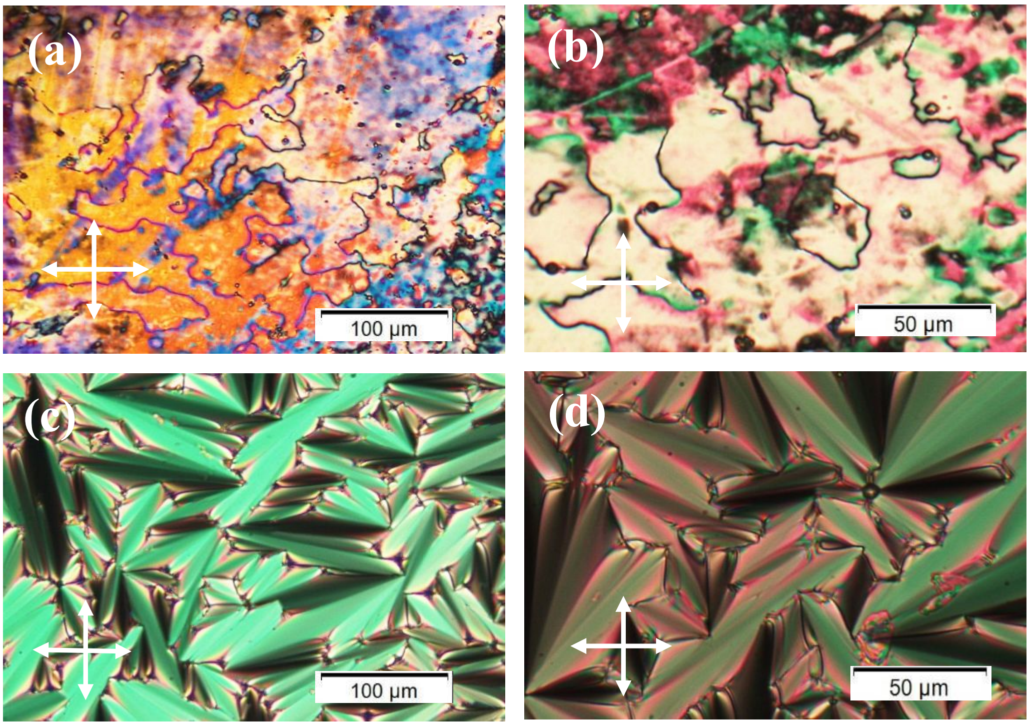


**Figure S5.** Polarizing optical microscope images of the mesophase textures obtained for the homogeneously (planarly) aligned sample: (a) & (b) marble textures of the N phase (T =130.1

°C and 126.2 °C); (c) & (d) the focal-conic fan texture of the SmA phase (T = and 120.3 °C and 114.1 °C).

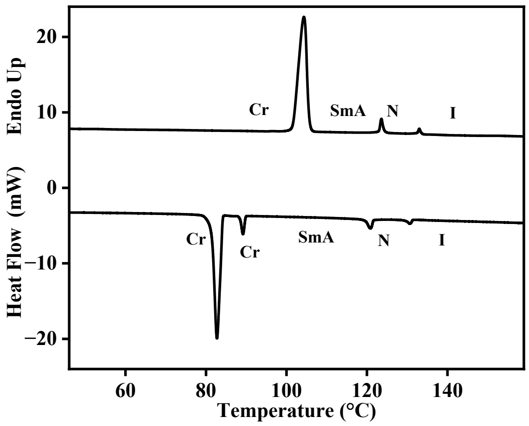


**Figure S6.** DSC thermograms of sample recorded for the first heating-cooling cycle at a rate of 5 K/min.

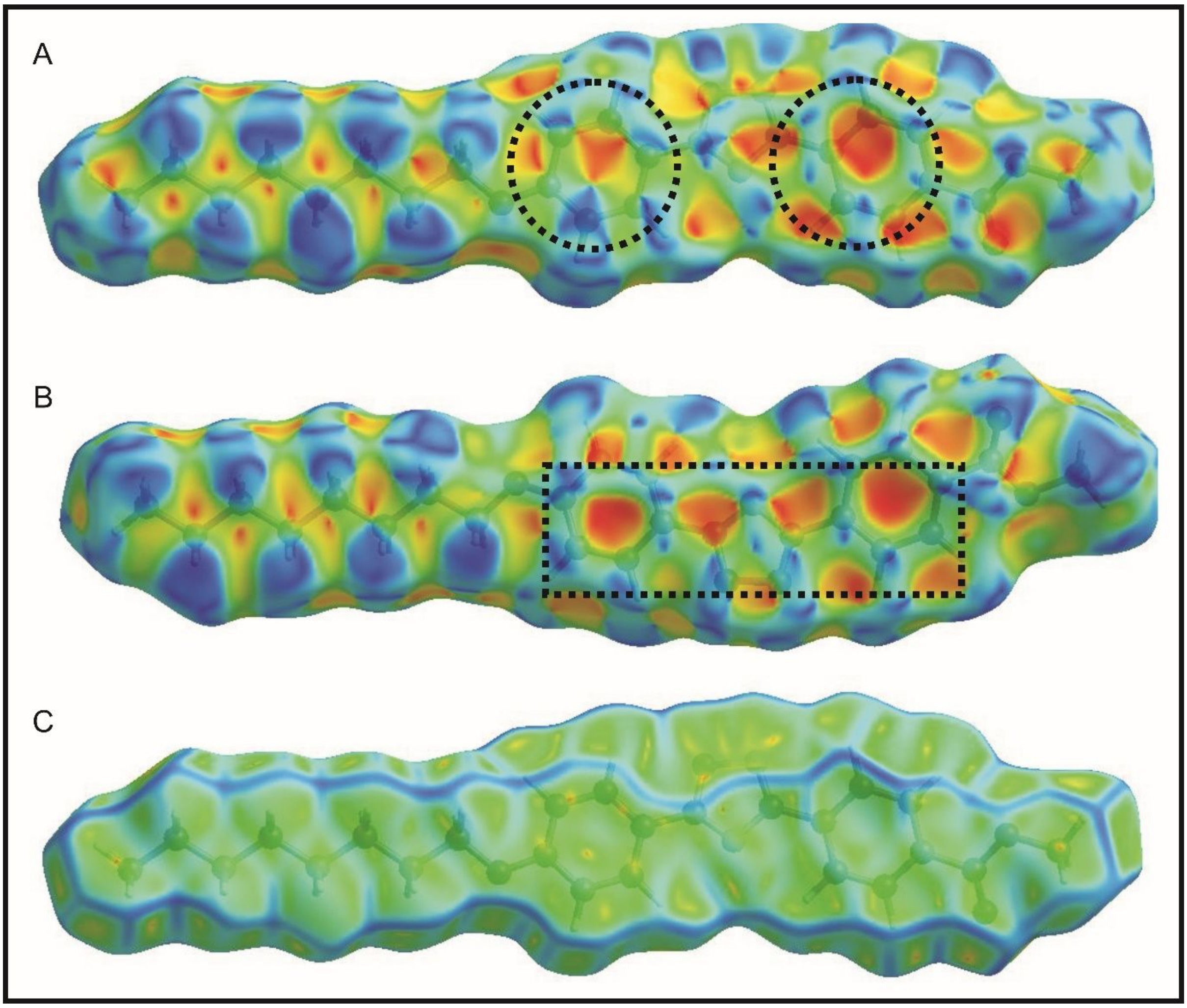

**Figure S7. Molecular surface analysis of the title compound.** The molecular surfaces are depicted as follows: (A-B) shape index provide a 180° rotated view, offering a comprehensive visualization from multiple perspectives, and (C) curvedness of the title compound.

**Table S1.** Fractional atomic coordinates ($\times 10^4$) and equivalent isotropic displacement parameters ($Å^2 \times 10^3$). $U_{eq}$ is defined as 1/3 of the trace of the orthogonalised $U_{IJ}$ tensor.

| Atom | *x* | *y* | *z* | U(eq) |
|---|---|---|---|---|
| O1 | 12668.3(19) | 3765.1(15) | 565.5(4) | 73.5(3) |
| O3 | 10738(2) | 8128.4(15) | 3715.3(4) | 75.2(3) |
| O4 | 2526.3(19) | 7102.0(15) | 5500.1(4) | 73.6(3) |
| O2 | 8736(2) | 3129(2) | 391.8(6) | 102.3(5) |
| N2 | 7865(2) | 6125.9(16) | 3220.7(5) | 63.6(3) |
| N1 | 11742(3) | 7813.5(19) | 3199.2(6) | 74.8(4) |
| C11 | 6973(3) | 7092.7(18) | 4174.6(6) | 59.5(4) |
| C4 | 12519(3) | 5491.7(19) | 1586.9(6) | 62.7(4) |
| C2 | 10485(3) | 3734(2) | 703.6(7) | 66.5(4) |
| C3 | 10449(3) | 4510.0(18) | 1281.2(6) | 58.8(4) |
| C10 | 8445(3) | 7061.9(18) | 3692.3(6) | 60.3(4) |
| C6 | 10166(3) | 5893.6(18) | 2363.9(6) | 57.0(3) |
| C14 | 4083(3) | 7147.7(19) | 5082.7(6) | 61.9(4) |
| C9 | 9974(3) | 6627.8(18) | 2932.4(6) | 58.4(3) |
| C16 | 4563(3) | 6097(2) | 4147.4(7) | 67.9(4) |
| C15 | 3136(3) | 6121(2) | 4595.9(7) | 71.1(4) |
| C8 | 8248(3) | 4231(2) | 1519.2(7) | 68.0(4) |
| C7 | 8105(3) | 4899(2) | 2058.4(7) | 65.7(4) |
| C5 | 12385(3) | 6193(2) | 2123.5(7) | 64.3(4) |
| C20 | -217(3) | 8659(2) | 7319.6(7) | 67.2(4) |
| C18 | 1286(3) | 7867(2) | 6389.9(7) | 68.4(4) |
| C17 | 3393(3) | 8095(2) | 6023.6(7) | 70.7(4) |
| C21 | 358(3) | 9649(2) | 7890.5(7) | 68.4(4) |
| C1 | 12819(4) | 2995(3) | 8.9(7) | 85.5(5) |
| C13 | 6488(3) | 8112(2) | 5119.7(7) | 78.9(5) |
| C22 | -1831(3) | 9541(2) | 8225.7(7) | 70.8(4) |
| C19 | 1947(3) | 8836(2) | 6965.0(7) | 70.4(4) |
| C12 | 7920(3) | 8066(2) | 4669.6(7) | 79.2(5) |
| C23 | -1292(3) | 10523(2) | 8796.9(7) | 76.7(5) |
| C24 | -3509(4) | 10483(3) | 9110.5(8) | 93.3(6) |

**Table S2.** Anisotropic displacement parameters ($Å^2 \times 10^3$). The anisotropic displacement factor exponent takes the form: $-2\pi^2[h^2a^{*2}U_{11}+2hka^*b^*U_{12}+\ldots]$.

| Atom | $U_{11}$ | $U_{22}$ | $U_{33}$ | $U_{12}$ | $U_{13}$ | $U_{23}$ |
|---|---|---|---|---|---|---|
| O1 | 64.4(6) | 95.0(8) | 62.9(6) | 30.6(6) | 10.9(5) | -11.6(5) |
| O3 | 66.2(7) | 85.4(7) | 61.5(6) | 9.9(5) | 12.7(5) | -9.2(5) |
| O4 | 61.2(6) | 91.6(8) | 59.8(6) | 16.0(5) | 11.3(5) | -11.8(5) |
| O2 | 68.6(8) | 148.6(13) | 82.0(8) | 32.2(8) | -2.4(6) | -36.5(8) |
| N2 | 62.1(7) | 65.2(7) | 62.2(7) | 19.9(6) | 11.7(6) | -4.3(6) |
| N1 | 69.3(8) | 86.2(9) | 61.0(7) | 15.8(7) | 15.1(6) | -4.9(7) |
| C11 | 62.2(8) | 60.7(8) | 54.6(8) | 19.6(6) | 7.4(6) | -1.6(6) |
| C4 | 52.0(8) | 73.7(9) | 64.0(8) | 23.1(7) | 10.6(6) | 0.3(7) |
| C2 | 61.2(9) | 74.1(9) | 64.1(9) | 23.0(7) | 8.1(7) | -3.5(7) |
| C3 | 56.7(8) | 61.8(8) | 59.9(8) | 22.9(6) | 8.1(6) | -0.5(6) |
| C10 | 61.8(8) | 59.8(8) | 57.6(8) | 18.2(6) | 8.1(6) | 0.9(6) |
| C6 | 59.3(8) | 59.1(8) | 56.8(8) | 24.9(6) | 9.0(6) | 3.8(6) |
| C14 | 60.3(8) | 69.0(9) | 55.2(8) | 20.3(7) | 7.7(6) | -2.1(6) |
| C9 | 61.4(8) | 60.8(8) | 56.0(8) | 23.9(6) | 8.8(6) | 4.5(6) |
| C16 | 63.8(9) | 74.8(9) | 58.5(8) | 16.5(7) | 3.9(7) | -12.5(7) |
| C15 | 57.3(9) | 79.7(10) | 67.2(9) | 12.6(7) | 6.3(7) | -10.6(8) |
| C8 | 53.1(8) | 77.1(10) | 70.4(9) | 19.0(7) | 5.5(7) | -9.8(7) |
| C7 | 53.8(8) | 74.8(9) | 68.6(9) | 22.0(7) | 12.5(7) | -4.0(7) |
| C5 | 55.0(8) | 73.2(9) | 63.3(8) | 20.4(7) | 4.9(6) | -3.9(7) |
| C20 | 71.9(10) | 69.8(9) | 60.0(8) | 24.2(7) | 9.1(7) | -2.4(7) |
| C18 | 67.1(9) | 77.9(10) | 60.0(9) | 24.6(8) | 9.5(7) | -2.7(7) |
| C17 | 67.2(9) | 82.3(10) | 58.3(9) | 20.9(8) | 4.3(7) | -8.6(7) |
| C21 | 74.9(10) | 71.9(9) | 58.8(8) | 25.6(8) | 7.0(7) | -0.5(7) |
| C1 | 85.8(12) | 107.3(14) | 63.9(10) | 35.3(10) | 14.9(8) | -16.8(9) |
| C13 | 67.1(10) | 92.8(12) | 60.7(9) | 7.9(8) | 9.8(7) | -17.3(8) |
| C22 | 76.5(10) | 71.7(9) | 61.8(9) | 22.3(8) | 9.6(8) | -4.9(7) |
| C19 | 71.9(10) | 78.3(10) | 60.2(9) | 24.2(8) | 7.7(7) | -1.1(7) |
| C12 | 62.7(9) | 89.8(11) | 69.0(10) | 6.3(8) | 10.8(7) | -13.5(8) |
| C23 | 90.1(12) | 77.6(10) | 61.5(9) | 28.1(9) | 7.8(8) | -6.8(8) |
| C24 | 104.4(14) | 98.1(13) | 73.7(11) | 30.5(11) | 19.9(10) | -13.8(10) |

**Table S3.** Hydrogen atom coordinates (Å×$10^4$) and isotropic displacement parameters (Å$^2$×$10^3$).

| Atom | x | y | z | U(eq) |
|---|---|---|---|---|
| H4 | 14006(3) | 5680.7(19) | 1430.5(6) | 75.3(5) |
| H16 | 3910(3) | 5407(2) | 3822.0(7) | 81.5(5) |
| H15 | 1527(3) | 5445(2) | 4573.0(7) | 85.4(5) |
| H8 | 6851(3) | 3587(2) | 1314.0(7) | 81.6(5) |
| H7 | 6617(3) | 4681(2) | 2217.5(7) | 78.9(5) |
| H5 | 13779(3) | 6864(2) | 2323.9(7) | 77.2(5) |
| H20a | -998(3) | 7470(2) | 7383.7(7) | 80.7(5) |
| H20b | -1347(3) | 9034(2) | 7104.0(7) | 80.7(5) |
| H18a | 113(3) | 8241(2) | 6190.6(7) | 82.0(5) |
| H18b | 540(3) | 6671(2) | 6449.1(7) | 82.0(5) |
| H17a | 4582(3) | 7719(2) | 6214.3(7) | 84.8(5) |
| H17b | 4131(3) | 9281(2) | 5948.3(7) | 84.8(5) |
| H21a | 1412(3) | 9228(2) | 8115.6(7) | 82.1(5) |
| H21b | 1219(3) | 10829(2) | 7828.6(7) | 82.1(5) |
| H1a | 12360(30) | 3612(11) | -280.9(7) | 128.2(8) |
| H1b | 14436(7) | 3024(17) | -42(3) | 128.2(8) |
| H1c | 11760(20) | 1841(6) | -18(2) | 128.2(8) |
| H13 | 7142(3) | 8793(2) | 5446.9(7) | 94.6(6) |
| H22a | -2885(3) | 9959(2) | 7999.2(7) | 85.0(5) |
| H22b | -2691(3) | 8360(2) | 8286.4(7) | 85.0(5) |
| H19a | 2758(3) | 10026(2) | 6906.9(7) | 84.5(5) |
| H19b | 3058(3) | 8425(2) | 7172.8(7) | 84.5(5) |
| H12 | 9547(3) | 8698(2) | 4700.1(7) | 95.0(6) |
| H23a | -320(3) | 10061(2) | 9033.0(7) | 92.0(6) |
| H23b | -354(3) | 11693(2) | 8739.7(7) | 92.0(6) |
| H24a | -4463(15) | 10966(18) | 8885(3) | 139.9(9) |
| H24b | -4431(15) | 9332(3) | 9178(6) | 139.9(9) |
| H24c | -3038(4) | 11126(17) | 9468(3) | 139.9(9) |

**Table S4.** Bond lengths of the title compound and bond lengths extracted from CSD studies, presented with mean, median, minimum, maximum, and number of hits.

| Atom/s | | Current Study | CSD Studies | | | | |
|---|---|---|---|---|---|---|---|
| | | Length/Å | Mean/Å | Minimum/Å | Maximum/Å | Median | No. of hits |
| O1 | C2 | 1.333(2) | 1.329(23) | 0.897 | 1.737 | 1.332 | 8903 |
| O1 | C1 | 1.452(2) | 1.448(23) | 1.023 | 1.808 | 1.447 | 11029 |
| O3 | N1 | 1.419(2) | 1.419(18) | 1.296 | 1.613 | 1.417 | 2546 |
| O3 | C10 | 1.351(2) | 1.344(08) | 1.320 | 1.363 | 1.345 | 153 |
| O4 | C14 | 1.358(2) | 1.372(23) | 1.028 | 1.687 | 1.371 | 10248 |
| O4 | C17 | 1.436(2) | 1.432(33) | 0.708 | 1.824 | 1.434 | 10446 |
| O2 | C2 | 1.201(2) | 1.223(28) | 0.897 | 1.618 | 1.222 | 11016 |
| N2 | C10 | 1.299(2) | 1.297(14) | 1.248 | 1.405 | 1.296 | 488 |
| N2 | C9 | 1.384(2) | 1.368(21) | 1.228 | 1.448 | 1.365 | 1531 |
| N1 | C9 | 1.300(2) | 1.304(15) | 1.255 | 1.480 | 1.304 | 292 |
| C11 | C10 | 1.454(2) | 1.472(02) | 1.245 | 1.595 | 1.472 | 7018 |
| C11 | C16 | 1.389(2) | 1.390(16) | 0.898 | 1.688 | 1.391 | 19134 |
| C11 | C12 | 1.382(2) | 1.390(16) | 0.898 | 1.688 | 1.391 | 19134 |
| C4 | C3 | 1.386(2) | 1.390(16) | 0.898 | 1.688 | 1.391 | 19134 |
| C4 | C5 | 1.384(2) | 1.384(18) | 0.882 | 1.639 | 1.384 | 20000 |
| C2 | C3 | 1.484(2) | 1.495(22) | 1.042 | 1.832 | 1.495 | 10532 |
| C3 | C8 | 1.383(2) | 1.390(16) | 0.898 | 1.688 | 1.391 | 19134 |
| C6 | C9 | 1.471(2) | 1.471(16) | 1.375 | 1.666 | 1.470 | 2386 |
| C6 | C7 | 1.385(2) | 1.390(16) | 0.898 | 1.688 | 1.391 | 19134 |
| C6 | C5 | 1.393(2) | 1.390(16) | 0.898 | 1.688 | 1.391 | 19134 |
| C14 | C15 | 1.386(2) | 1.383(19) | 0.921 | 1.622 | 1.384 | 13237 |
| C14 | C13 | 1.380(2) | 1.383(19) | 0.921 | 1.622 | 1.384 | 13237 |
| C16 | C15 | 1.373(2) | 1.384(18) | 0.882 | 1.639 | 1.384 | 20000 |
| C8 | C7 | 1.380(2) | 1.384(18) | 0.882 | 1.639 | 1.384 | 20000 |
| C20 | C21 | 1.515(2) | 1.513(53) | 0.348 | 2.092 | 1.520 | 12799 |
| C20 | C19 | 1.517(2) | 1.513(53) | 0.348 | 2.092 | 1.520 | 12799 |
| C18 | C17 | 1.499(2) | 1.506(41) | 1.006 | 1.962 | 1.509 | 10323 |
| C18 | C19 | 1.518(2) | 1.513(53) | 0.348 | 2.092 | 1.520 | 12799 |
| C21 | C22 | 1.514(2) | 1.513(53) | 0.348 | 2.092 | 1.520 | 12799 |
| C13 | C12 | 1.382(2) | 1.384(18) | 0.882 | 1.639 | 1.384 | 20000 |
| C22 | C23 | 1.512(2) | 1.511(65) | 0.733 | 2.079 | 1.519 | 11932 |
| C23 | C24 | 1.514(2) | 1.498(74) | 0.495 | 2.071 | 1.513 | 12326 |

**Table S5.** Bond angles of the title compound and bond angles extracted from CSD studies, presented with mean, median, minimum, maximum, and number of hits.

| | | | Current Study | CSD Studies | | | | |
|---|---|---|---|---|---|---|---|---|
| Atom/s | | | Angle/° | Mean/° | Minimum/° | Maximum/° | Median/° | No. of hits |
| C1 | O1 | C2 | 115.73(13) | 115.80(1.95) | 84.996 | 139.256 | 115.843 | 8903 |
| C10 | O3 | N1 | 106.37(11) | 106.07(0.67) | 101.879 | 108.139 | 106.062 | 153 |
| C17 | O4 | C14 | 119.19(12) | 117.95(2.59) | 82.032 | 156.943 | 117.88 | 10082 |
| C9 | N2 | C10 | 102.81(12) | 102.75(0.69) | 99.661 | 104.449 | 102.715 | 72 |
| C9 | N1 | O3 | 103.22(12) | 103.28(0.61) | 100.081 | 106.626 | 103.261 | 287 |
| C16 | C11 | C10 | 119.76(13) | 120.56(1.51) | 81.862 | 131.873 | 120.554 | 10182 |
| C12 | C11 | C10 | 121.66(14) | 120.56(1.51) | 81.862 | 131.873 | 120.554 | 10182 |
| C12 | C11 | C16 | 118.57(14) | 118.57 (1.39) | 56.113 | 145.393 | 118.606 | 14263 |
| C5 | C4 | C3 | 120.39(13) | 120.80(1.16) | 89.761 | 135.754 | 120.785 | 14049 |
| O2 | C2 | O1 | 123.12(15) | 123.44 (1.95) | 86.107 | 151.442 | 123.502 | 8903 |
| C3 | C2 | O1 | 112.98(13) | 112.31(1.49) | 92.646 | 141.378 | 112.27 | 4284 |
| C3 | C2 | O2 | 123.90(14) | 122.06(3.26) | 68.367 | 145.921 | 123.065 | 10535 |
| C2 | C3 | C4 | 122.70(13) | 120.38 (2.22) | 89.996 | 143.15 | 120.536 | 10700 |
| C8 | C3 | C4 | 119.29(14) | 118.57(1.39) | 56.113 | 145.393 | 118.606 | 14263 |
| C8 | C3 | C2 | 118.01(14) | 120.38 (2.22) | 89.996 | 143.15 | 120.536 | 10700 |
| N2 | C10 | O3 | 112.84(12) | 113. 30(0.84) | 110.066 | 119.111 | 113.359 | 153 |
| C11 | C10 | O3 | 118.44(13) | 117.89 (2.10) | 105.408 | 122.041 | 117.989 | 96 |
| C11 | C10 | N2 | 128.72(14) | 127.81 (2.13) | 115.924 | 140.056 | 128.154 | 384 |
| C7 | C6 | C9 | 119.55(13) | 120.75(1.48) | 94.526 | 141.678 | 120.661 | 4025 |
| C5 | C6 | C9 | 121.20(14) | 120.75 (1.48) | 94.526 | 141.678 | 120.661 | 4025 |
| C5 | C6 | C7 | 119.24(14) | 118.57(1.39) | 56.113 | 145.393 | 118.606 | 14263 |
| C15 | C14 | O4 | 116.01(13) | 119.99(4.92) | 88.013 | 146.813 | 118.864 | 10390 |
| C13 | C14 | O4 | 124.46(14) | 119.99(4.92) | 88.013 | 146.813 | 118.864 | 10390 |
| C13 | C14 | C15 | 119.52(14) | 120.19(1.46) | 87.555 | 142.557 | 119.99 | 11408 |
| N1 | C9 | N2 | 114.75(13) | 114.47(0.72) | 112.336 | 117.745 | 114.449 | 287 |
| C6 | C9 | N2 | 122.22(14) | 122.94(2.07) | 109.661 | 149.096 | 123.034 | 1207 |
| C6 | C9 | N1 | 123.02(13) | 122.22(0.88) | 118.379 | 124.262 | 122.303 | 248 |
| C15 | C16 | C11 | 120.68(14) | 120.80(1.16) | 89.761 | 135.754 | 120.785 | 14049 |
| C16 | C15 | C14 | 120.30(14) | 119.72(1.15) | 89.677 | 132.676 | 119.764 | 12469 |
| C7 | C8 | C3 | 120.61(14) | 120.80(1.16) | 89.761 | 135.754 | 120.785 | 14049 |
| C8 | C7 | C6 | 120.33(13) | 120.80(1.16) | 89.761 | 135.754 | 120.785 | 14049 |
| C6 | C5 | C4 | 120.12(14) | 120.80(1.16) | 89.761 | 135.754 | 120.785 | 14049 |
| C19 | C20 | C21 | 114.46(14) | 114.33(4.99) | 60.033 | 180 | 113.93 | 11885 |
| C19 | C18 | C17 | 113.40(14) | 113.47(4.39) | 83.839 | 170.766 | 113.287 | 9682 |
| C18 | C17 | O4 | 107.61(13) | 108.30(3.70) | 80.042 | 168.829 | 107.814 | 10211 |
| C22 | C21 | C20 | 113.91(14) | 114.33(4.99) | 60.033 | 180.00 | 113.93 | 11885 |
| C12 | C13 | C14 | 119.85(15) | 119.72(1.15) | 89.677 | 132.676 | 119.764 | 12469 |
| C23 | C22 | C21 | 114.65(14) | 115.23(8.76) | 21.595 | 180.00 | 114.075 | 10756 |
| C18 | C19 | C20 | 112.78(14) | 114.33(4.99) | 60.033 | 180 | 113.93 | 11885 |
| C13 | C12 | C11 | 121.01(16) | 120.80(1.16) | 89.761 | 135.754 | 120.785 | 14049 |
| C24 | C23 | C22 | 113.85(16) | 113.45(7.19) | 28.671 | 179.932 | 113.083 | 11932 |

**Table S6.** Torsion angles of the title compound.

| A | B | C | D | Angle/° | | A | B | C | D | Angle/° |
|---|---|---|---|---|---|---|---|---|---|---|
| O1 | C2 | C3 | C4 | 13.62(17) | | N2 | C9 | C6 | C7 | 11.93(16) |
| O1 | C2 | C3 | C8 | -166.32(14) | | N2 | C9 | C6 | C5 | -168.70(14) |
| O3 | N1 | C9 | N2 | 0.46(13) | | N1 | C9 | C6 | C7 | -166.81(16) |
| O3 | N1 | C9 | C6 | 179.28(10) | | N1 | C9 | C6 | C5 | 12.56(18) |
| O3 | C10 | N2 | C9 | 1.10(14) | | C11 | C16 | C15 | C14 | 0.25(19) |
| O3 | C10 | C11 | C16 | 175.53(14) | | C11 | C12 | C13 | C14 | 1.5(2) |
| O3 | C10 | C11 | C12 | -5.89(18) | | C4 | C3 | C8 | C7 | -0.92(17) |
| O4 | C14 | C15 | C16 | 179.16(14) | | C4 | C5 | C6 | C9 | -179.78(14) |
| O4 | C14 | C13 | C12 | 179.92(18) | | C4 | C5 | C6 | C7 | -0.41(17) |
| O4 | C17 | C18 | C19 | 179.26(13) | | C2 | C3 | C8 | C7 | 179.03(15) |
| O2 | C2 | C3 | C4 | -166.75(18) | | C3 | C8 | C7 | C6 | 1.49(18) |
| O2 | C2 | C3 | C8 | 13.3(2) | | C20 | C21 | C22 | C23 | -179.96(15) |
| N2 | C10 | C11 | C16 | -4.32(19) | | C20 | C19 | C18 | C17 | 177.32(15) |
| N2 | C10 | C11 | C12 | 174.26(18) | | C21 | C22 | C23 | C24 | 176.67(16) |

**Table S7.** Conformational analysis of phenyl octyloxy moiety of the title compound with CSD structures. The table shows the dihedral angles of phenyl octyloxy moiety of different conformations. There are nine major types of conformations identified with several subgroups. The subgroups are marked as a, b, c, etc. and for each group the dihedral angles extreme two structures are given. Here, SP represents synperiplanar, SC represents synclinal, AP represents antiperiplanar, and AC represents anticlinal. The torsion angle with clockwise rotation of the bond, by convention, was considered positive and anti-clockwise rotation was considered negative. Dihedral angles for miscellaneous subtypes (3c, 5d, 6c, 7c, and 9h) are not shown due to complexity. The number of structures for each group are given in brackets.

| Group | | Refcode | 1 | 2 | 3 | 4 | 5 | 6 | 7 | 8 |
|---|---|---|---|---|---|---|---|---|---|---|
| | | Dihedral angle | C13-C14-O4-C17 | C14-O4-C17-C18 | O4-C17-C18-C19 | C17-C18-C19-C20 | C18-C19-C20-C21 | C19-C20-C21-C22 | C20-C21-C22-C23 | C21-C22-C23-C24 |
| | | Current Study | 178.2 | 177.3 | 179.3 | 177.3 | -178.3 | 176.7 | -180.0 | 176.6 |
| | | | AP | AP | AP | AP | AP | AP | AP | AP |
| Type 1 (226) | | OLOXAP | -166.4 | 158.5 | -179.9 | 164.5 | -179.5 | 168.3 | 176.4 | 168.4 |
| | | | AP | AP | AP | AP | AP | AP | AP | AP |
| | | UWACIF_mol3 | 175.5 | -173.8 | -179.6 | -177.2 | 175.2 | -172.1 | 174.6 | -175.5 |
| | | | AP | AP | AP | AP | AP | AP | AP | AP |
| Type 2 (7) | 2a (3) | WIRXOK01_mol3 | 174.4 | -176.1 | 175.9 | -178.1 | 177.9 | -179.4 | 171.0 | 68.3 |
| | | | AP | AP | AP | AP | AP | AP | AP | SC |
| | | JESZIR_mol4 | 174.9 | -176.0 | 177.4 | -178.4 | 177.4 | -179.6 | 170.4 | 67.2 |
| | | | AP | AP | AP | AP | AP | AP | AP | SC |
| | 2b (4) | GEQWEI | 174.0 | -171.2 | -177.7 | -179.3 | -175.8 | 179.9 | -174.5 | -65.4 |
| | | | AP | AP | AP | AP | AP | AP | AP | -SC |
| | | SUSQAY01 | -170.1 | 168.5 | 178.9 | -180.0 | -179.3 | -178.2 | -176.9 | -62.0 |
| | | | AP | AP | AP | AP | AP | AP | AP | -SC |
| Type 3 (6) | 3a (2) | GIYWAP02_mol2 | 173.3 | -172.6 | -180.0 | -174.4 | 173.9 | -178.7 | -70.9 | -177.6 |
| | | | AP | AP | AP | AP | AP | AP | -SC | AP |
| | | TECMAQ | -177.6 | -177.9 | -179.8 | 174.9 | 179.7 | -179.4 | -70.9 | 166.3 |
| | | | AP | AP | AP | AP | AP | AP | -SC | AP |
| | 3b (2) | JOKBAP_mol1 | -174.3 | -179.9 | -174.4 | 176.1 | -175.6 | 175.0 | 60.6 | 164.0 |
| | | | AP | AP | AP | AP | AP | AP | SC | AP |
| | | XOCXAC | -179.5 | 178.7 | -177.8 | 179.8 | 178.2 | 178.3 | 65.8 | 177.1 |
| | | | AP | AP | AP | AP | AP | AP | SC | AP |
| Type 4 (5) | (3) | KOSZIB | -178.3 | 177.8 | -178.9 | 170.8 | 174.3 | 57.7 | 179.0 | -179.2 |
| | | | AP | AP | AP | AP | AP | SC | AP | AP |
| | | KAGVIB_mol4 | 173.5 | -168.8 | -175.0 | 174.3 | -173.6 | 67.4 | 165.1 | 175.2 |
| | | | AP | AP | AP | AP | AP | SC | AP | AP |
| Type 5 (15) | 5a (4) | TIFLIE_mol2 | 175.5 | -175.2 | -172.0 | -174.0 | -56.6 | -164.6 | -178.1 | -173.9 |
| | | | AP | AP | AP | AP | -SC | AP | AP | AP |
| | | YIZFEV_mol1 | -178.8 | 175.7 | 174.1 | 174.1 | -75.0 | -177.4 | 177.1 | 180.0 |
| | | | AP | AP | AP | AP | -SC | AP | AP | AP |
| | 5b (2) | ODUZIA_mol1 | -177.3 | 177.9 | -179.9 | -178.0 | 82.6 | 178.1 | -170.5 | 176.4 |
| | | | AP | AP | AP | AP | SC | AP | AP | AP |
| | | AHIQOZ | -178.5 | -179.6 | -177.9 | -178.8 | 72.5 | 174.5 | 175.9 | -177.7 |

| | | | | | | | | | | |
|---|---|---|---|---|---|---|---|---|---|---|
| | | | AP | AP | AP | AP | SC | AP | AP | AP |
| | 5c (2) | REHMAS | -174.4 | 176.8 | -179.0 | 177.6 | 70.9 | 177.7 | 178.0 | 60.5 |
| | | | AP | AP | AP | AP | SC | AP | AP | SC |
| | | REHMEW | -177.7 | 179.1 | 170.9 | 177.9 | 84.3 | 166.5 | 178.4 | 78.2 |
| | | | AP | AP | AP | AP | SC | AP | AP | SC |
| Type 6 (6) | 6a (2) | NOCSUU_mol3 | -178.0 | -178.9 | 178.9 | 66.3 | 168.3 | 162.5 | 169.6 | 167.2 |
| | | | AP | AP | AP | SC | AP | AP | AP | AP |
| | | YUMYEM_mol1 | -176.6 | 176.7 | -175.4 | 70.1 | 177.2 | -177.5 | 178.6 | -176.0 |
| | | | AP | AP | AP | SC | AP | AP | AP | AP |
| | 6b (3) | YUMYEM_mol2 | 177.3 | -178.8 | 175.6 | -69.8 | -178.3 | -177.9 | 178.5 | -179.7 |
| | | | AP | AP | AP | -SC | AP | AP | AP | AP |
| | | NOCSUU_mol1 | 178.0 | 178.9 | -178.9 | -66.3 | -168.3 | -162.4 | -169.6 | -167.2 |
| | | | AP | AP | AP | -SC | AP | AP | AP | AP |
| Type 7 (109) | 7a (30) | REFJAN_mol2 | 167.1 | -165.9 | -69.7 | -176.4 | -178.6 | -175.7 | -177.9 | -179.1 |
| | | | AP | AP | -SC | AP | AP | AP | AP | AP |
| | | OVOGAK_mol2 | 169.7 | -178.9 | 60.0 | -154.6 | 178.7 | -174.7 | -174.9 | 179.4 |
| | | | AP | AP | SC | AP | AP | AP | AP | AP |
| | 7b (33) | KAPCIO_mol4 | 172.8 | -166.3 | 66.4 | -165.2 | 179.8 | -177.8 | -175.9 | 176.3 |
| | | | AP | AP | SC | AP | AP | AP | AP | AP |
| | | REFJAN_mol1 | -167.2 | 165.8 | 69.8 | 176.4 | 178.6 | 175.7 | 177.8 | 179.1 |
| | | | AP | AP | SC | AP | AP | AP | AP | AP |
| Type 8 (7) | (7) | YEBZUB_mol2 | -157.7 | 70.9 | 61.4 | 177.3 | -178.1 | 174.8 | -177.0 | -177.3 |
| | | | AP | SC | SC | AP | AP | AP | AP | AP |
| | | BASDUX | 173.4 | -76.7 | -176.5 | -176.7 | -67.4 | 176.1 | -179.8 | -67.9 |
| | | | AP | -SC | AP | AP | -SC | AP | AP | -SC |
| Type 9 (38) | 9a (5) | HANXAV_mol2 | -88.8 | 169.7 | -172.4 | 168.8 | -175.0 | 178.9 | -176.5 | -177.0 |
| | | | -SC | AP | AP | AP | AP | AP | AP | AP |
| | | HANXAV_mol1 | -92.9 | -176.3 | 171.7 | -177.5 | 174.1 | -174.8 | 172.4 | -179.5 |
| | | | -AC | AP | AP | AP | AP | AP | AP | AP |
| | 9b (12) | LAYREL_mol2 | 95.8 | 163.9 | -169.0 | 171.7 | -170.3 | 169.0 | -178.6 | 175.1 |
| | | | AC | AP | AP | AP | AP | AP | AP | AP |
| | | ELOYAH_mol1 | 86.2 | 178.6 | 175.7 | 179.1 | 175.8 | 173.8 | -179.2 | -178.3 |
| | | | SC | AP | AP | AP | AP | AP | AP | AP |
| | 9c (4) | TAHZUZ_mol2 | -100.0 | 158.4 | 59.1 | 163.5 | 172.7 | 175.0 | 177.1 | 177.8 |
| | | | -AC | AP | SC | AP | AP | AP | AP | AP |
| | | COMWEJ | 91.4 | 179.5 | 66.5 | 176.5 | 178.5 | -178.1 | -171.2 | -179.9 |
| | | | AC | AP | SC | AP | AP | AP | AP | AP |
| | 9d (2) | COMXOU_mol2 | -84.1 | 164.9 | -64.9 | 179.6 | -176.4 | -177.1 | -179.3 | -177.6 |
| | | | -SC | AP | -SC | AP | AP | AP | AP | AP |
| | | UCIVOU_mol1 | -92.9 | 167.2 | -72.5 | -178.3 | 179.8 | -178.6 | 178.0 | -174.3 |
| | | | -AC | AP | -SC | AP | AP | AP | AP | AP |
| | 9e (2) | COMXOU_mol1 | 101.3 | 166.2 | -67.3 | -177.7 | -176.4 | 179.0 | -171.1 | -178.9 |
| | | | AC | AP | -SC | AP | AP | AP | AP | AP |
| | | UCIVOU_mol3 | 96.9 | 165.1 | -66.4 | -174.8 | -177.9 | -162.4 | -155.0 | -171.1 |
| | | | AC | AP | -SC | AP | AP | AP | AP | AP |
| | 9f (4) | EGOFAJ | 72.8 | 177.8 | -68.2 | -171.1 | 174.8 | -179.3 | -180.0 | -176.7 |
| | | | SC | AP | -SC | AP | AP | AP | AP | AP |

| | | | | | | | | | | |
|---|---|---|---|---|---|---|---|---|---|---|
| | | NAPJOE_mol1 | 93.4 | -166.2 | -68.5 | -173.7 | 169.5 | -168.0 | -178.5 | -165.2 |
| | | | AC | AP | -SC | AP | AP | AP | AP | AP |
| | 9g (3) | LAYREL_mol1 | 91.3 | -175.5 | 65.7 | -177.2 | -178.4 | -178.4 | -178.9 | -179.2 |
| | | | AC | AP | SC | AP | AP | AP | AP | AP |
| | | UCIVOU_mol2 | 80.2 | -166.6 | 89.0 | -176.6 | -147.3 | -173.8 | 177.3 | -176.9 |
| | | | SC | AP | SC | AP | -AC | AP | AP | AP |

**Table S8.** Molecular pairs and interaction energies (kJ/mole) acquired from the energy framework calculations for title compund. N represents the number of pairs, and R the distance between molecular centroids (Å). $E_{tot} = k_{ele}E_{ele} + k_{pol}E_{pol} + k_{disp}E_{disp} + k_{rep}E_{rep}$, wherein $E_{ele}$, $E_{pol}$, $E_{disp}$, and $E_{rep}$ represent electrostatic, polarization, dispersion, and exchange-repulsion energy components.

| N | Symop | R | Electron Density | $E_{ele}$ | $E_{pol}$ | $E_{dis}$ | $E_{rep}$ | $E_{tot}$ |
|---|---|---|---|---|---|---|---|---|
| 1 | -x, -y, -z | 8.29 | B3LYP/6-31G(d,p) | -13.0 | -2.4 | -47.6 | 31.7 | -37.4 |
| 1 | x, y, z | 5.92 | B3LYP/6-31G(d,p) | -7.3 | -2.7 | -54.3 | 26.3 | -40.7 |
| 1 | x, y, z | 26.11 | B3LYP/6-31G(d,p) | -5.3 | -1.5 | -9.5 | 0.0 | -14.9 |
| 1 | -x, -y, -z | 5.12 | B3LYP/6-31G(d,p) | -12.8 | -2.3 | -108.8 | 45.9 | -81.7 |
| 1 | -x, -y, -z | 7.14 | B3LYP/6-31G(d,p) | -14.1 | -2.5 | -96.4 | 41.4 | -75.1 |

**Table S9.** Grid box dimensions for the molecular targets selected for docking the standard antimicrobial compounds and the title compound.

| Organism | PDB id | x size (Å) | y size (Å) | z size (Å) | x centre | y centre | z centre |
|---|---|---|---|---|---|---|---|
| *E. coli* | 1HNJ | 20 | 28 | 18 | 27.049 | 12.305 | 32.323 |
| | 4KFG | 12 | 10 | 12 | 21.403 | 24.358 | -3.211 |
| *S. typhi* | 5E68 | 32 | 22 | 28 | 2.505 | 2.108 | -0.945 |
| *S. aureus* | 5ZH8 | 14 | 22 | 22 | 15.748 | 45.473 | 12.696 |
| *S. mutans* | 3AIE | 24 | 20 | 24 | 189.421 | 50.269 | 193.477 |
| | 4TQX | 18 | 14 | 30 | 8.965 | 24.444 | -11.272 |
| *C. albicans* | 4LEB | 28 | 28 | 20 | 4.368 | 4.955 | -18.211 |
| *T. brucei* | 4MW2 | 22 | 18 | 32 | 2.251 | 23.756 | 21.031 |
